# Group singing through the lens of polyvagal theory: A pilot study in patients with Parkinson’s disease

**DOI:** 10.1371/journal.pone.0337210

**Published:** 2025-12-30

**Authors:** Elke Wünnenberg, Nicola Baumann

**Affiliations:** 1 University of Trier, Trier, Germany; 2 International Initiative Singing Hospitals, Stuttgart, Germany; University Hospital Eriangen at Friedrich-Alexander-University Erlangen-Numberg, GERMANY

## Abstract

There is a need to counteract the chronic progression of Parkinson’s disease (PD). This complex challenge requires new theoretical frameworks and practical strategies. In this study, we implemented group singing in line with the Singing Hospitals format and examined it through the lens of Polyvagal Theory to evaluate its potential benefits for people with PD. According to Polyvagal Theory, facilitating a shift in autonomic states from defense to safety and social engagement may enhance physiological regulation and thereby promote well-being across physical, mental, emotional, and social dimensions. In a pilot study, we recruited patients with PD and examined their responses to two group singing formats (weekly sessions: *N1* = 13, one-day-workshop: *N*2 = 14) and a non-singing control group (*N*3 = 22). We designed scales of physical, mental, emotional, and social states that PD patients rated before and after eight one-hour singing sessions (*N*1) or an eight-hour singing workshop (*N*2). Findings show that PD patients benefited from group singing across both settings (large effect size: *d* = 2.43). Furthermore, self-reported interoceptive sensibility, used as a proxy for polyvagal autonomic reactivity, showed a substantial reduction in discomfort after weekly singing, while remaining constant in the control group (large effect size: *d* = 0.94). We observed the predicted singing-dependent effects and interpreted them as a shift in autonomic regulation consistent with Polyvagal Theory. Singing may represent a feasible, low-threshold resource for coping. Furthermore, Polyvagal Theory may provide an innovative framework for PD and help to bridge motor and non-motor symptoms.

## Introduction

“*Singing is an elixir of life*” (Adamek, 1996)

The sociologist, psychologist, and musician Karl Adamek [1] pointed out that expression through singing is existential to human beings. In his studies, he emphasized the importance of singing as self-help in everyday life. Kang and colleagues [2] attribute a therapeutic potential to singing in their review but criticize that conceptualization of the underlying cause-effect relationships is lacking. The aim of the nonprofit initiative *Singing Hospitals* is to integrate singing as a resource and a powerful tool for self-help and coping into the healthcare system [3–8]. In 2009, Singing Hospitals formed an international multidisciplinary network to facilitate group singing formats for patients with physical or psychological diseases in health care facilities and train singing leaders for these diverse settings.

The concept of *Singing Hospitals* [3–6] comprises an experience-oriented form of group singing that goes beyond traditional choral singing. The shift from a performance-oriented approach to singing to one that emphasizes authentic, non-judgmental self-expression in a collaborative and creative group setting brings the practice of singing closer to the principles of music therapy. In this context, singing becomes not just an act of outward display but one that fosters both individual growth and interpersonal relationships. Singers sit or stand in a circle without sheet music, warm up their bodies and voices, move, dance, and interact through musically supported forms of encounter (unison, canon, call and response, spontaneous polyphony, etc.).

Over the past 15 years, this form of singing has been applied with a wide range of patients including patients with Parkinson’s disease, chronic obstructive pulmonary disease, stroke, dementia, and psychosomatic and psychiatric conditions. The practice-based observations with this form of singing have been generally positive. However, systematic empirical research is lacking. So far, there is only a qualitative evaluation of this concept of singing [6]. Group singing is conceptualized here as a co-regulator that promotes coping by fostering systemic balance across physiological and psychological domains [3–8].

**Fig 1:**
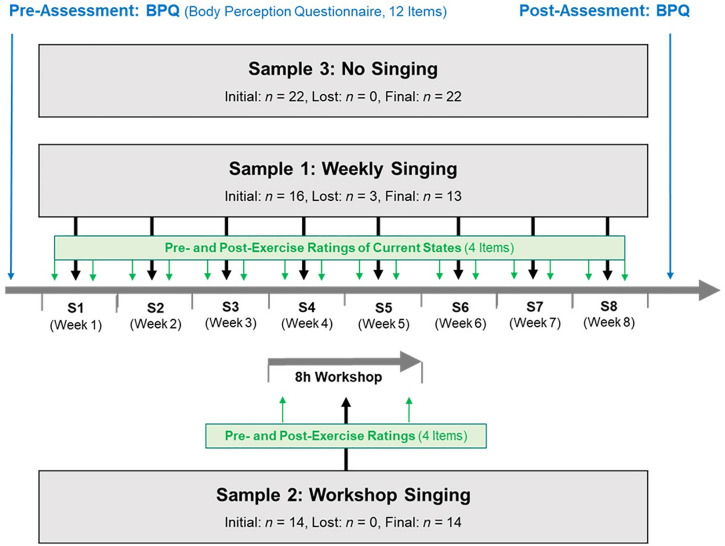
CONSORT flowchart. Overview of the study design, procedure, and measures.

In this pilot study, we implemented group singing in line with the Singing Hospitals format and examined it through the lens of Polyvagal Theory to evaluate its potential benefits for people with PD [9–19]. Our pilot study aimed to: (a) describe the singing processes in a more integrative way; (b) test the measurement instruments in preparation for a randomized controlled design study; (c) examine the suitability of the selected singing approach and its adaptation in the field of PD; and (d) develop recommendations for treatment and self-help approaches for people with PD (for an overview of the study design, procedure, and measures, see Fig 1).

### The need for coping with Parkinson’s disease

Developing new theoretical frameworks and testing innovative therapeutic approaches is a priority according to the *International Parkinson and Movement Disorder Society* [20]. Due to the lack of curative approaches, strategies for coping and neuroprotection and personalized management of PD are called for [21,22]. In addition to the chronic degenerative course, the extremely high complexity of PD is a major challenge for medicine. PD was recently conceptualized as a complex neuropsychiatric disorder [23], including emotion (i.e., depression and anxiety), perception and thinking (i.e., psychosis), and motivation (i.e., impulse control disorders and apathy). Multiple non-motor symptoms often precede the leading motor symptoms, sometimes by several years. Patients rate these non-motor symptoms as an even greater burden than the motor symptoms [24]. By reviewing the clinical characteristics and neural substrates of various symptoms (negative effects on gait, handwriting, grip, and speech) and highlighting how PD and its medical and surgical treatments effect motor symptoms, Moustafa and colleagues [25], offered a “unified framework” explaining the range of motor symptoms in PD. They argued that various motor symptoms in PD “reflect dysfunction of neural structures responsible for action selection, motor sequencing, and coordination and execution of movement” (p.727) [25]. However, an integrative understanding of motor and non-motor symptoms is currently not possible.

Prenger and colleagues [26] report that PD leads to a variety of emotional and communicative changes disrupting social functioning. These would include problems producing emotional facial expressions (i.e., facial masking) and emotional speech (i.e., dysarthria) as well as recognizing verbal and nonverbal emotional cues from others. A previous systematic mixed methods review [27] described how PD symptoms (e.g., tremor, facial masking, and neuropsychiatric symptoms) compromise relationships (e.g., partnership, friends, and family) and lead to early retirement. Moreover, they pointed out that conventional PD therapy rarely has a positive impact on the functioning of social roles. According to Prenger and colleagues, social symptoms of PD can result in “severe negative consequences, including stigma, dehumanization, and loneliness, which might affect the quality of life to an even greater extent than more well-recognized motor or cognitive symptoms” (p. 1) [26]. So far, dealing with PD has continued to present new challenges for clinicians and practitioners. The disorder appears to gain in complexity, making the search for a linear, causal approach to overcoming it increasingly unlikely.

### Singing with patients of Parkinson’s disease

Di Benedetto and colleagues started to investigate a voice and choral singing treatment to improve speech and voice disorders in PD with “an amusing, agreeable, and collective approach” (p. 13) [28]. Semi-structured interviews revealed that singing was helpful to PD and stroke patients. Singing supported patients’ coping with disease burden, including social isolation, low mood, and communication difficulties [29].

Stegemöller and colleagues [30–33] explored the benefits of singing on a range of symptoms experienced by people with PD. They began by recording voice, respiratory, and quality-of-life measures before and after eight weeks of singing [30] and examined the acute effects of one hour of group therapeutic singing on physiological measures of stress [32] and clinical motor symptoms [32,33] in patients with PD. They found significant improvements in maximum inspiratory and expiratory pressures and phonation time when singing. Notably, both voice-related and overall quality of life improved in patients [30]. In addition, their study showed a significant decrease in mean scores for gait, posture, and tremor, but not for speech and facial expression, or bradykinesia [33]. Contrary to expectations, they found no significant changes in cortisol levels or motor improvement, nor any significant correlation between both variables in PD patients in a single session [32]. However, the singing group showed a tendency to feel less sad compared to the control group. In addition, the heart rate increased in the singing group and decreased in the control group after the session. From this single session, Stegemöller and colleagues [32] conclude that singing without sheet music, accompanied by vocal exercises, is a promising yet exploratory intervention for people with PD. Tamplin’s team went further and developed an interdisciplinary *ParkinSong* model, comprising high-effort vocal and respiratory tasks, speech exercises, group singing, and social communication opportunities. Results from a controlled trial with active control condition at two dosage levels (weekly vs. monthly) over three months [34], followed by a four-armed controlled clinical trial over 12 months [35], demonstrated promising potential to counteract deterioration in voice, speech, respiratory strength, voice-related quality-of-life outcomes and well-being from mild to moderate PD. In a randomized crossover study, Butala and colleagues [36] found that weekly group singing may improve conversational voice volume and aspects of quality of life, including emotional well-being and body discomfort, in people with PD, although baseline group differences limited attribution of effects solely to singing.

Moreover, Yeo and colleagues [37] investigated the effects of individual singing-induced swallowing exercises in patients with advanced PD in comparison to a matched usual care control group. Therapeutic singing exercises did not improve but preserved swallowing function in advanced PD patients compared to the controls and improved swallowing-related quality of life in those with advanced-stage diseases. There is a first hint that an online format of singing is practicable and appreciated by patients of PD but more thought needs to be given to the delivery format of group singing programs in the context of telemedicine [38,39].

In their review of the current state of studies, Barnish and colleagues [40] cite significant methodological limitations, such as a lack of control groups. However, in their assessment, the studies suggest a “broader benefit” of singing with effects on functional communication, cognitive status, motor function, and quality of life that should be evaluated through further studies. Besides singing, dancing is becoming of greater interest to serve as an integrative therapeutic intervention “via music, metaphorical language, and a socially reinforced reality of art-partaking” (p.1) [41]. The interest in performing arts as a therapeutic medium in PD is increasing [42].

### Polyvagal theory as a new leading concept for coping

**Fig 2:**
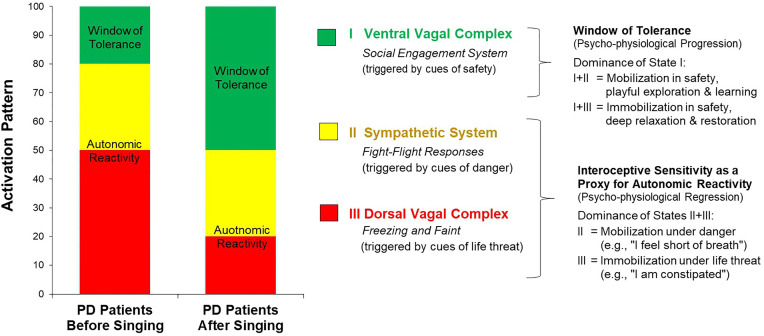
Activation pattern of the autonomic nervous system before and after a singing intervention with patients of Parkinson’s disease (PD), according to the Polyvagal Theory from SW Porges [10–13]. Note: I Ventral Vagal Complex: a state in which one *feels safe*, takes part in social interaction, and feels connected with oneself and with others *(including social engagement system);* II Sympathetic System: a state of feeling danger in which *mobilization* occurs. People show fight or flight responses, that is, they enact active defensive (*fight-flight responses*); III Dorsal Vagal Complex: a state in which people experience life threat, *immobilization* in form of a shutdown as a passive defense (*freezing, fainting)*.

We refer to Polyvagal Theory by Porges [9–19] as an integrative framework for this study. Porges postulated a neurophysiological model describing neural circuits involved in the regulation of autonomic states and how these, in turn, shape adaptive behavior and social engagement (e.g., the face-heart connection [10,17–19], gut-brain interactions [43]). Within this framework, the autonomic nervous system is conceptualized as a phylogenetically ordered hierarchy comprising the ventral vagal complex (State I, see Fig 2) the sympathetic system (State II, see Fig 2), and the dorsal vagal complex (State III, see Fig 2). These subsystems support, respectively, social engagement and restoration (I), mobilization in fight/flight responses (II), and immobilization with freezing and shutdown in the face of extreme threat (III). Ontogeny is proposed to mirror phylogeny, such that postnatally these systems mature in the order of the hierarchy, from dorsal to ventral. In line with Jacksonian dissolution [10,17–19], acute threat, illness, or injury is hypothesized to trigger hierarchical deactivation from the newest to the oldest systems.

It is important to note that the autonomic state functions as an intervening variable that mediates physiological regulation, behavioral expression, emotional reactivity, and health outcomes. Central to this model is the notion that the face–heart connection allows social interactions to regulate visceral states [10,17–19] and that gut–brain interactions provide an additional pathway linking autonomic regulation to subjective experience [43]. Signals of safety, danger, and life threat from within the body and from the external environment may trigger different autonomic states through a process termed “neuroception” [10,17–19]. Consequently, each autonomic state alters an individual’s capacity to feel safe and to flexibly navigate between defensive modes and states of connection (see Fig 2, [10,17–19]). Consistent with this view, previous studies have shown that disruptions in the regulation of autonomic states are associated with hyperarousal, fatigue, and a range of psychosomatic and psychiatric symptoms [44–46].

As hierarchically organized, the evolutionary later developed systems constraint the preceding ones, so that the ventral vagal complex (State I, see Fig 2) has the most important effect on health. When it overrides the older, more basal systems, individuals may operate within a broader window of tolerance [47,48], characterized by well-being, coping capacity, and resilience [10–13,17–19].

Why and how might a singing format strengthen states of the ventral vagal complex? The *Singing Hospitals* format is designed to foster perceived safety, shared attention, and coordinated action through music and interpersonal encounter among participants [3–6]. Thus, singing may stimulate the ventral vagal complex both directly—via neurophysiological effectors [13,14,17–19]—and indirectly, by creating a socially reinforced context that enables bidirectional signaling and co-regulation in line with the hierarchical model of autonomic regulation [15–19,49,50]. Ventral vagal complex activity is associated with rapid, flexible cardiac regulation, which can be facilitated by prolonged, slow exhalation phases characteristic of singing. Beyond its roles in ingestion and orienting, the complex is hypothesized to regulate state-dependent social behaviors, including nursing, bonding, vocal communication, and facial prosody. Special visceral efferent components of cranial nerves (V, VII, IX, X, and XI) are thought to coordinate facial expression, vocal prosody, head–neck orientation, posture, and middle-ear muscle tone, thereby functionally linking social communication with autonomic regulation, named social engagement system [10,17–19,50]. Accordingly, there are multiple ways in which singing could modulate participants’ states (see subscales of the self-developed rating scale):

- **From physical rigidity to flexibility (subscale 1):** through deeper diaphragmatic breathing with prolonged exhalation, tension-releasing vocal resonance, alongside music-induced axial rotation and postural realignment;
- **From mental dullness to clarity (subscale 2):** by shifting hyper-/hypoarousal toward socially engaged attention, and by using structured rhythm and phrasing for re-orientation, present-moment focus, and sustained attention;
- **From being emotionally uninvolved to being touched (subscale 3):** through self-congruent lyrics and vocal prosody that deepen self-expression and support affect regulation and embodied processing (e.g., via tempo, rhythm, and dynamics);
- **From social isolation to connection (subscale 4):** through experiences of togetherness fostered by synchronization, shared attention, and co-creation among participants.

Taken together, the *Singing Hospitals* format may function as a multi-domain regulatory resource—simultaneously supporting autonomic regulation, emotional expression, attentional focus, and social connectedness—and thereby help individuals with PD to cope more effectively with everyday challenges.

Within Polyvagal Theory, the state-dependent responsiveness of the autonomic nervous system to cues of safety, danger, and life threat is called “autonomic reactivity” by Cabrera and colleagues [51]. They developed a self-report questionnaire to look into these states by seeking key sensations of autonomic reactivity. In contrast to interoceptive accuracy, this questionnaire captures people’s interoceptive sensibility as “an individual’s beliefs about their interoceptive ability and the degree to which they feel engaged by interoceptive signals” (p. 66) [51]. Interoception comprises the process by which individuals’ sense, interpret, and integrate signals from within the body, thus detecting their current state across conscious and unconscious levels [52]. Enhancing individuals’ awareness of and capacity to regulate their autonomic state in response to signals of safety, fight-flight, and freezing is getting a key role in therapy. Thus, reinforcing interoception abilities is a relevant focus for clinical interventions [53]. Furthermore, vagal nerve stimulation is proposed as a method to promote calming, enhance social engagement, and support physiological balance through activation of the ventral vagal pathway [54]. According to Polyvagal Theory, all resource-oriented, preventive and supportive formats—such as self-help or coping—should also aim to foster patient’s awareness of these states [54].

In this pilot study, we refer to Polyvagal Theory to talk about how singing in the format of *Singing Hospitals* interacts with autonomic states. We expected that a weekly singing group activity (one hour per week for eight weeks) would positively affect PD patients’ well-being during the singing sessions (H1). Furthermore, we explored whether this would be a reproducible effect within another context, expecting that a singing group activity in the format of an eight-hour workshop would have similar positive effects on PD patients (H2). Finally, we assumed that singing could have more long-lasting effects on stress regulation through repeated stimulation of the social engagement system by the singing activity. Thus, we expected the weekly singing intervention to reduce patients’ body awareness of autonomous reactivity compared to a no singing control group (H3).

## Methods

### Participants and procedure

The study was approved by the Ethics Committee of the University of Trier (Germany) in May 28^th^ 2019 (EK 04/2019). We did not determine our sample size in advance but collected data from as many patients as possible from June 18^th^ 2019 until November 30^th^ 2019. Study data are available online: https://pasa.psycharchives.org/reviewonly/45567bb6ba636cc5943e6ccd48d5222c757322bb8942c57f6f07267b9634cbc8. We confirm that all ongoing and related trials for this intervention are registered at the *German Clinical Trials Register* (DRKS-IDDRKS00035851). Since we missed the prospective registration of clinical trials as a prerequisite for publication in leading medical journals, we registered our pilot study during the submission process. All participants included in the study provided written informed consent. There were no activities to increase compliance. Participants continued their current PD medication and concomitant therapies and were recruited for two different single-arm feasibility studies (Fig 1).

#### Sample 1: Weekly singing intervention.

We recruited 16 patients (5 women, 11 men) diagnosed with PD (Hoehn and Yahr stages 1–2 [55]), who were willing to take part in a one-hour group singing activity per week over the course of 8 weeks (no masking). All participants had taken part in individual and group courses on self-directed training in PD, multiple sclerosis, and polyneuropathy with the sports scientist during the last two years. Two men left the singing group because they could not adapt to the form of singing used; one man dropped out in the fifth session due to a clinic stay. The final Sample 1 consisted of *N* = 13 PD patients (5 women, 8 men), aged 53–83 years (*M* = 65.77, *SD* = 8.39). Before (pre) and after (post) each of the eight singing sessions, participants rated their current state on a self-developed rating system described below (see Fig 1). In addition, they rated their body awareness on a standardized questionnaire at the beginning (T1) and after 2–3 months (T2) at the end of the pilot study.

In the eight singing sessions, the first author guided patients as a singing facilitator with a professional background in psychotherapy and music education. In accordance with the suggestion of the Singing Hospitals format [3–6], musically concise songs with life-affirming lyrics from various traditions and cultures were used. In adaptation for PD patients, we selected rhythmically accentuated songs with variable tempi to invite dancing steps and rotational movements, for example through the English-language nursery rhyme and a popular children’s song “Row, row, row the boat gently down the stream—merrily, merrily, merrily; life is just a dream.” The groups sang the selected songs repeatedly.

#### Sample 2: Workshop singing intervention.

We recruited a total of 14 PD patients, consisting of 11 women and 3 men, aged between 50 and 71 years (*M* = 62.53, *SD* = 6.33), by self-selection (without masking) during a meeting in presence of the self-help online group of *PARKINSonLIE e.V.* to which the first author was invited to give a group singing workshop. Sample 2 participants rated their current state at the beginning (pre) and end (post) of a one-day workshop of eight hours of singing using the same self-developed rating scales as in Sample 1 (Fig 1). In the workshop, we used a singing activity in accordance with the format of S*inging Hospitals* as described above. The workshop was organized as a self-help meeting.

#### Sample 3: Non-singing control group.

We recruited *N* = 22 PD patients (18 women, 4 men), aged 44–76 years (*M* = 60.09, *SD* = 8.07) by self-selection without masking within the self-help patient initiatives *PARKINSonLINE e.V.* and *Parkinson bewegt e*.V. Sample 3 participants completed the questionnaire online assessing body awareness and autonomic reactivity at the beginning (T1) and end (T2) of the pilot study (see Fig 1). As a control group, these PD patients did not participate in a singing activity.

### Materials

#### Self-developed rating scales.

A change-sensitive measurement tool was lacking to adequately capture the holistic effects of a potential basal shift at the level of the nervous system. Therefore, to evaluate the effects of group singing on participants’ current states, we developed a bipolar rating scale as feedback to the singing experience covering four dimensions: physical state (rigid vs. flexible), mental state (dull vs. clear), emotional state (uninvolved vs. touched), social state (isolated vs. connected). In the first two sessions, participants rated these dimensions on a scale from −3 to +3. Because of ceiling effects, we extended the scale range from −4 to +4 in sessions 3–8. Therefore, we did not include Sessions 1 and 2 in our analysis. Furthermore, not all participants attended every session (four patients missed session 4, two session 5, two different patients session 6, and four session 7). The mean absenteeism rate was 11.54%. To avoid missing data, we aggregated ratings across two sessions (3–4, 5–6, 7–8).

#### Body awareness, interoceptive sensibility and autonomic reactivity.

We used the 12-item very short form of the *Body Perception Questionnaire* (BPQ; [51,56]) as a proxy for interoceptive sensitivity and autonomic reactivity. Participants rate the extent to which they are aware of their autonomic states, excluding items that were “noisy” due to assumed extra-autonomous influences (e.g., “clumsiness or bumping into people” [56]. We used body awareness as a proxy for interoceptive sensibility and autonomic reactivity to evaluate the effects of singing (vs. non-singing) over time. In the present study, internal consistency was sufficient (Cronbach’s α = .82 among the *N* = 35 PD patients across Samples 1 and 3).

## Results

### Descriptive statistics

According to the norms of the 12-item Body Awareness Very Short Form [51,56], at the beginning of the pilot study, our samples of PD patients reported scores within a normal range. In the weekly singing group (*N* = 13), the body awareness of *M* = 27.92 (*SD* = 9.12) corresponds to a *T-*value of 51.5 [51], or 50.6 [56]. In the non-singing control group (*N* = 22), the body awareness of *M* = 26.64 (SD = 7.77) corresponds to a *T-*value of 50.7 [51] or 49.7 [56].

### Statistical analyses

To test our hypotheses, we planned to conduct three types of analyses of variance (ANOVA) using SPSS.14/IBM. First, in Sample 1, we planned to use 2 (time) x 3 (sessions) ANOVAs to test the effects of hourly singing across time (within: pre vs. post) and sessions (within: 3–4 vs. 5–6 vs. 7–8) on physical flexibility, clarity of mind, emotional involvement, and social connectedness, respectively. Second, in Samples 1 and 2, we planned to use a 2 (time) x 2 (group) ANOVA to test the effect of singing across time (within: pre vs. post) and group (between: weekly singing vs. workshop singing) on well-being (i.e., the mean across the four ratings mentioned above). Third, in Samples 1 and 3, we planned to use a 2 (time) x 2 (group) ANOVA on autonomic reactivity to test the effect of singing across time (within: pre vs. post) and group (between: weekly singing vs. no singing).

### Effects of weekly singing

**Fig 3:**
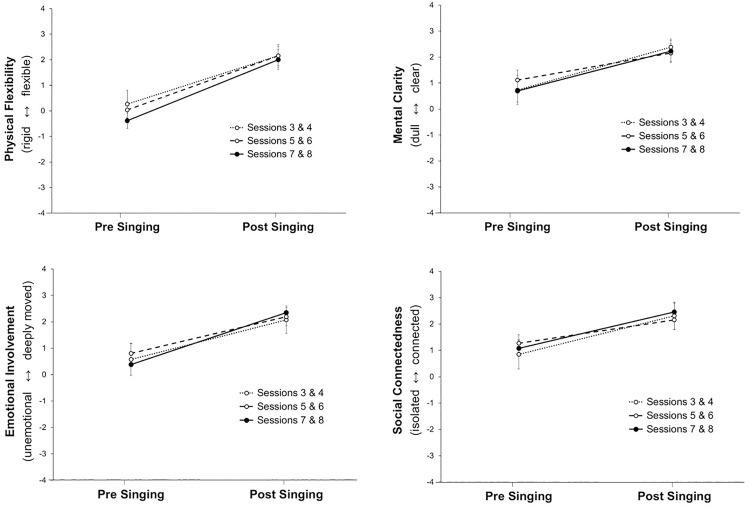
Means (and standard errors) of self-reported states pre- and post-one-hour long weekly singing activities among patients with Parkinson’s disease (*N* = 13).

To examine psychological changes during the weekly singing group activity, we conducted four separate 2 (time: pre vs. post treatment) x 3 (sessions: 3–4, 5–6, and 7–8) ANOVAs with repeated measures on both factors. Fig 3 illustrates the results. On all four dimensions of our self-developed rating scale, there were highly significant main effects of Time (Physical: *F*(1, 12) = 69.06, *p* < .001, $\eta_{p}^{\text{2}}$ =.852; Mental: *F*(1, 12) = 18.83, *p* < .001, $\eta_{p}^{\text{2}}$ =.611; Emotional: *F*(1, 12) = 25.41, *p* < .001, $\eta_{p}^{\text{2}}$ =.679; Social: *F*(1, 12) = 15.15, *p* < .002, $\eta_{p}^{\text{2}}$ =.558). Findings indicate that, across sessions, there were significant improvements from pre to post singing in patients’ self-rated physical flexibility (*M*_pre_ = 0.03 vs. *M*_post_ = 2.10, *p* < .001), clarity of mind (*M*_pre_ = 0.85 vs. *M*_post_ = 2.26, *p* < .001), emotional involvement (*M*_pre_ = 0.59 vs. *M*_post_ = 2.21, *p* < .001), and social connectedness (*M*_pre_ = 1.06 vs. *M*_post_ = 2.31, *p* < .002). On all four dimensions, there was no significant main effect of Session (all *F*s(2, 24) < 0.74, *p* > .490, $\eta_{p}^{\text{2}}$ <.058) indicating no continuous improvements from early to later sessions. Furthermore, on all four dimensions, there was no significant Time x Session interaction (all *F*s(2, 24) = 1.18, *p* < .323, $\eta_{p}^{\text{2}}$ <.090) indicating that the improvement through singing did not change over the course of the weekly sessions but was visible right from the start (i.e., session 3–4) and did not fade out towards the end (i.e., session 7–8). Taken together, the hourly singing activity improved not only mental clarity, social connectedness, and emotional involvement, but also patients’ physical flexibility.

### Weekly singing versus workshop singing

**Fig 4:**
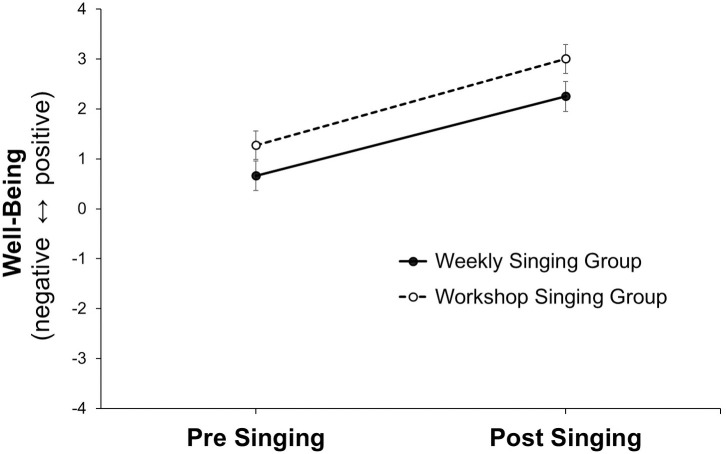
Means (and standard errors) of well-being (physical flexibility, mental clarity, emotional involvement, social connectedness) pre- and post-singing among patients with Parkinson’s disease in a weekly singing group (Sample 1, *N* = 13) and a workshop singing group (Sample 2, *N* = 14).

To explore if PD patients showed the same positive effects when singing was practiced in a different format, we conducted a 2 (time: pre, post) x 2 (group: weekly singing, workshop singing) ANOVA with repeated measures on the first factor. In Sample 1, we aggregated ratings across sessions 3–8 and across dimensions. In Sample 2, we aggregated ratings across dimensions. Again, there was a highly significant main effect of Time, *F*(1, 25) = 31.63, *p* < .001, $\eta_{p}^{\text{2}}$ =.596 (i.e., *d* = 2.429; see https://www.escal.site/) indicating significant improvements from pre to post singing in patients’ physical flexibility, mental clarity, emotional involvement, and social connectedness (*M*_pre_ = 1.12, *SD*_pre_ = 1.25 vs. *M*_post_ = 2.65, *SD*_pre_ = 1.13, *p* < .001). There was a significant main effect of Group, *F*(1, 25) = 6.59, *p* = .017, $\eta_{p}^{\text{2}}$ =.208, but no significant Time x Group interaction, *F*(1, 25) = 0.72, *p* = .791, $\eta_{p}^{\text{2}}$ =.003. Thus, patients of PD benefited from the two different singing activity settings in the same way (see Fig 4). A posthoc sample size calculation (ANOVA, repeated measures, within factors, effect size *f* = 1.215, *α* = .025, *N* = 27, 2 groups, 2 measurements, *r* = .50) indicated that we achieved a power (1 – *β*) = 1.00 for detecting a main effect of time.

### Weekly singing versus no singing

**Fig 5:**
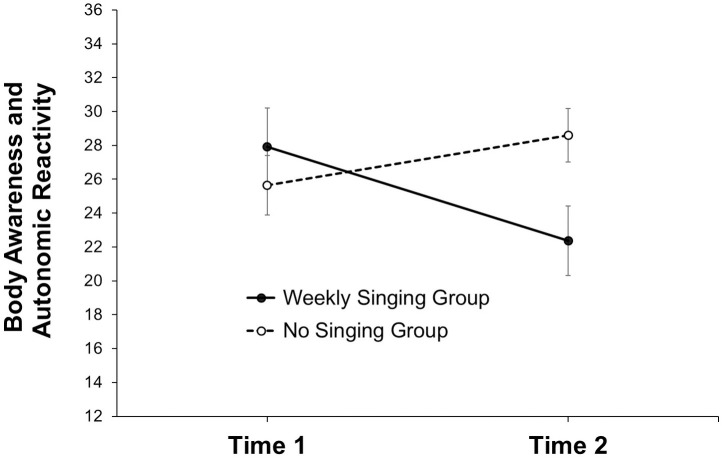
Means (and standard errors) in body perception and autonomic reactivity at baseline (Time 1) and after eight weeks (Time 2) among patients with Parkinson’s disease in a weekly singing group (Sample 1, *N* = 13) and a no singing control group (Sample 3, *N* = 22).

To test whether the weekly singing group activity reduced autonomic reactivity compared to a control group without singing, we conducted 2 (time: T1, T2) x 2 (group: weekly singing group, no singing group) ANOVA with repeated measures on the first factor. There were no significant main effects of Time, *F*(1, 33) = 0.80, *p* = .378, $\eta_{p}^{\text{2}}$ <.024, and Group, *F*(1, 33) = 1.61, *p* = .214, $\eta_{p}^{\text{2}}$ <.046. More importantly, the Time x Group interaction was significant, *F*(1, 33) = 7.22, *p* = .011, $\eta_{p}^{\text{2}}$ =.180 (i.e., *d* = 0.937; see https://www.escal.site/). Fig 5 illustrates the interaction. Independent *t-*Tests revealed that, at the beginning of the intervention, the singing group (*M*_pre_ = 27.92, *SD*_pre_ = 9.19) did not differ in autonomic reactivity from the control group (*M*_pre_ = 26.64, *SD*_pre_ = 7.77), *t*(33) = 0.44, *p* = .661, Diff = 1.29, 95% CI [−4.63, 7.20]; after eight weeks of singing, the singing group (*M*_post_ = 22.38, *SD*_post_ = 4.25) showed lower body awareness compared to the control group (*M*_post_ = 29.41, *SD*_post_ = 8.61), *t*(33) = −2.74, *p* = .010; Diff = −7.02, 95% CI [−12.24, −1.81]. Dependent *t-*Tes*t*s revealed that the singing group’s decrease in the markers of body awareness was (marginally) significant, *t*(12) = 2.18, *p* = .050; Diff = 5.54, 95% CI [0.01, 11.09], whereas the control group’s increase was not significant, *t*(21) = −1.51, *p* = .147; Diff = −1.51, 95% CI [−6.60, 1.06]. A posthoc sample size calculation (ANOVA, repeated measures, within-between interaction, effect size *f* = 0.469, *α* = .025, *N* = 35, 2 groups, 2 measuremen*t*s, *r* = .30) indicated that we achieved a power (1 – *β*) =.98 for detec*t*ing a Time x Group interaction.

## Discussion

The present study addressed the question of whether the Polyvagal Theory of Porges might be suitable to discuss intervention approaches in PD and how singing contributes to coping [3–6,14,18,19,40,50]. We conducted two different singing activities (hourly singing sessions over eight weeks and an eight-hour singing workshop) and evaluated the physical, mental, emotional, and social states at the beginning and end of singing through a self-developed rating scale (Samples 1 and 2). Furthermore, we assessed body awareness as a proxy for interoceptive sensibility and autonomous reactivity at the beginning and after the eight weeks of singing (vs. non-singing) activity among PD patients (Samples 1 and 3).

### Effects in weekly singing and workshop singing groups

In line with what other studies have found [28–37,40–42], singing once a week improved physical functioning, including mood and social participation, however, these gains were typically transient, with participants returning to a lower level between sessions. Specifically, we introduced four dimensions (physical, mental, emotional, and social) in our ratings before and after the group singing activity (see Fig 3) to distinguish specific effects of singing from an overall effect. Our statistical analysis revealed a significant uniform improvement emerging across all four dimensions through singing. When comparing weekly singing with a one-day singing workshop, we found a similar singing effect across all four dimensions (Fig 4). This consistent finding across two different settings indicates a general effect of singing. The main effect of singing had a large effect size (*d* = 2.43). In summary, the present results reinforce the findings of the meta-analysis by Barnish and colleagues [40] who suggest that singing could make a valuable contribution to disease management in PD.

### Effects on interoceptive sensibility and autonomous reactivity through singing

Consistent with our hypothesis, the weekly group singing activity resulted in significantly lower scores in the 12-item Body Awareness Very Short Form compared to the control group (Fig 5). This means that patients with PD were less aware of body discomfort after the singing activity. Referring to the principles of Polyvagal Theory [14,18,50], we assume that our form of group singing—characterized among others by eye contact, deepened breathing, and an expanded range of vocalization—may foster an autonomic state associated with the social engagement system (Fig 2). According to Polyvagal Theory, this system provides the psychophysiological foundation for self-regulation, co-regulation through social interaction, and mutually cooperative behavior. In previous studies, the activity of the social engagement system promoted physiological processes and emotional states associated with coping and health [57,58]. Thereby, the stimulation of the social engagement system has led to positive effects, especially in the treatment of emotional trauma [59].

### Toward a model of singing effects according to Porges’ polyvagal theory

Our pilot study encourages further investigation into the extent to which group singing may facilitate a shift in autonomic state regulation toward ventral vagal activation and a physiological state of safety and social engagement, conducive to symptom reduction and enhanced well-being. At the end of the singing group, patients felt more physically flexible, mentally clearer, emotionally involved, and socially connected (Figs 3 and 4). How can we explain such a general shift through singing? We might gain insights from looking at research on dancing among patients with PD [60–62]. Koch and colleagues [62], for example, found that dancing positively influenced embodied identity. Singing might have a similar effect. In a review of medical and social science articles including group singing and rhythm in music as well as personal experience and reasoning, Buetow and colleagues [63] assume that Parkinson’s disease disrupts both social and biological rhythms. They suggest that participating in group singing may foster psychological states such as connectedness and flow, which could support rhythmic synchronization. This, in turn, may help alleviate motor timing difficulties and emotional processing challenges, ultimately enhancing quality of life.

It is important to note that in these previous explanations, dancing is treated as well as singing in an either/or manner: either as a primarily physical activity, thus focusing on body-related parameters or motor symptoms of PD, or as a form of psychosocial support, thus focusing on non-motor aspects such as quality of life. Only recently have there been initial attempts to take an integrative view. For example, Fontanesi and DeSouza [41] compared dance to an exercise intervention of matched intensity and found different outcomes indicating that dance entails intrinsic artistic elements—hypothesized to influence affective responses, experiences of beauty, self-efficacy, and gait performance—that are absent in exercise.

We propose that the concept of autonomic reactivity might contribute to describing the highly dysfunctional dynamics in symptom progression in PD. Consistent with Polyvagal Theory, signals of inner or outer threat might create those crucial boundary conditions leading to the cascade of motor, non-motor, and socioemotional symptoms as seen in PD. The advantage of our explanatory approach proposed here is that it could overcome this split between physical and social aspects in creative interventions such as dancing and singing. In general, embodied experience—including expressive gesture and movement, perceived social safety and connection, and emotional involvement within our singing format—may co-vary with psychophysiological balance and be paralleled by activation of the ventral vagal complex and the social engagement system; these processes are mutually reinforcing. Consistent with Polyvagal Theory, Porges emphasizes bottom-up influences whereby the autonomic state constrains or enables higher-order perception, attention, and volition. Rather than treating symptoms one by one, a polyvagal-informed approach targets the prevailing autonomic state, matching interventions to the individual’s momentary capacity for regulation, connection, and learning. Leveraging the body’s adaptive tendencies is intended to yield more enduring, intrinsic adaptive effects [18,19]. This aligns with the view that restoring dynamic homeostasis is a central goal of effective physiological regulation, as emphasized by Billman [64]. Accordingly, we highlight a close interconnection between physical and social processes that promote stress regulation and foster learning-oriented, cooperative, and restorative behavior.

Similar to trauma-exposed people, patients with Parkinson’s disease (PD) may show persistent alterations of autonomic balance and sensorimotor control that culminate in chronic autonomic dysregulation, expressed as hypervigilance, dissociative tendencies, anxiety, depression, and diverse somatic symptoms [18,19]. Within a polyvagal perspective, such patterns could reflect a long-standing shift toward defensive states—i.e., withdrawal of ventral vagal influence, recruitment of sympathetic mobilization, and dorsal vagal shutdown—even in the absence of immediate threat as a chronic dominance of the basal states (II + III, see Fig 2).

Several PD-specific mechanisms might maintain this anchoring. First, weakening of the social engagement system due to hypomimia and reduced vocal prosody may diminish cues of safety and interpersonal co-regulation. Second, attenuated orienting responses—for example restricted axial rotation and narrowed visual scanning (“tunnel vision”)—may limit environmental appraisal and the detection of safety signals. Third, impaired motor expression of arousal and startle—manifesting as festination and freezing—may hinder the resolution of defensive activation, thereby perpetuating state dysregulation.

To our knowledge, no PD studies have systematically operationalized the body therapy notion of “muscular armor” [65] or examined whether singing-based interventions can release such bracing patterns and thereby shift autonomic state toward ventral-vagal engagement. These hypotheses warrant targeted, state-aware investigation.

The earliest link between PD and the vagus nerve traces back to the original descriptions of Lewy-type inclusions in brainstem nuclei, including the dorsal motor nucleus of the vagus in 1912/1913 [66]. In later accounts, the vagus moved to the foreground with the Braak staging hypothesis from 2003 [67]. Recently, researchers studied the effects of stimulating the vagus nerve [68]. However, polyvagal assumptions regarding the hierarchical organization of the autonomic nervous system and the role of co-regulation have not yet been considered. Only in a brief essay in 2012, Diederich and Parent referenced Polyvagal Theory and suggested that symptoms such as impaired automatic gait or olfactory loss may reflect dysfunction in evolutionary ancient neural networks in PD [69], however, this hypothesis has not yet been systematically pursued in PD research.

We propose using Polyvagal Theory [9–19] to develop an integrative view on motor and non-motor symptoms in PD. Beside preventing degenerative nerve damage or substitute endogenous substances (e.g., dopamine), modulating psychophysiological states as described above could be an intervening variable whether symptoms escalate or decrease. The way autonomic states are organized in a hierarchy (see Fig 2) means that activating the social engagement system would constraint autonomic reactivity. Group singing could strengthen a highly adaptive self- and co-regulatory response to stressors from the inner and outer world. Conversely, chronic inactivation of the social engagement system could lead to a psychophysiological regression to basal systems with corresponding stress flooding and rigidification reactions, as observed in the course of PD. Singing could help to overcome this regression by strengthening the social engagement system. In general, it can be assumed that the reduction of autonomic reactivity in favor of the activity of the ventral vagal complex will lead to an alleviation of symptoms. Interoceptive sensitivity could help patients in gaining body information about which global affect state they are in and how this is changed by singing.

In other patient groups, researchers found a vicious cycle between autonomic dysregulation and chronic disease [70], autonomic reactivity and the buffering effects of adolescent friendships [71], social co-regulation of the autonomic nervous system between infants and their caregivers [72], atypical autonomic regulation and poor psychological well-being in college females with maltreatment histories [73], autonomic dysregulation and diabetes-relates distress [74], and cardiac autonomic dysregulation and joint hypermobility in adolescent with functional abdominal pain disorders [75]. To study coping with PD, we propose to refer to these insights, for example, the mutual influence of traumatic stress and the autonomic brain-gut connection. In this field, Polyvagal Theory is already used as an integrative framework for psychosocial and gastrointestinal pathology [43,76,77]. Here, singing was theorized to be suitable to cause a favorable “switch” in the system of the autonomic nervous system. In the following, we outline the theoretical and practical implications of a polyvagal perspective in coping with PD.

## Theoretical and practical implications

Consistent with the aims of our pilot study, the present findings lead to several implications. First, our findings suggest that the effects of singing can be described in a more integrative way. Neither a biological micro-perspective nor a global quality-of-life perspective on singing, as adopted in most previous studies [40], is appropriate. Second, our findings suggest that our instruments are (with slight adaptations) suitable for a randomized controlled study design. Future studies should also test whether longer singing intervals would lead to long-term effects on top of the short-term benefits observed here. A cost-benefit analysis could reveal optimal outcomes for patients with PD in clinical settings.

Third, Schiavio and Altenmüller [78] point to the potential of music-based approaches for PD as they can account for the interactions between cognition, body, and environment. They emphasize that linear cause-and-effect models cannot adequately represent the interwoven and fused nature of cognition, body, and environment. In this pilot study, we faced the interplay between cognition, body, and environment as realized in music and the complexity of PD by referring to the findings of Polyvagal Theory. Due to its theoretical foundation and its testable predictions, the selected group singing approach is suitable for adaptation in the field of PD.

Looking ahead, the treatment of Parkinson’s disease resembles Heracles’ task of confronting the many-headed Hydra in Greek mythology. Each time Heracles cut off one of Hydra’s heads, two new ones grew in its place, until he finally defeated the creature by cauterizing the neck to prevent regrowth. Similarly, Parkinson’s disease (PD) can be seen as a “multi-headed” challenge: when one symptom is managed, others may emerge or intensify. This metaphor illustrates the complexity of PD, in which motor and non-motor symptoms interact, and compensation in one domain may provoke dysregulation in another. Effective approaches may therefore require integrative rather than symptom-oriented strategies that address the underlying regulatory mechanisms rather than isolated manifestations—much like burning the Hydra’s necks instead of merely cutting off its heads. Therefore, we propose using Polyvagal Theory to understand the underlying causes in their complexity and interweaving of motor, non-motor, and psychosocial symptoms of PD and to evaluate treatments. Because this study introduces a polyvagal perspective into Parkinson’s research and, to our knowledge, illustrates it for the first time through a group singing intervention, the next section offers a critical appraisal of the limitations and potential of this approach.

## Limitations and future perspectives

The Polyvagal Theory [9–19] extends classical stress models (e.g., Siegel [47–49]) by introducing an evolutionary, hierarchical, and functional differentiation of the vagus nerve into ventral and dorsal vagal branches, each supporting distinct modes of autonomic regulation—adaptive and defensive states—with or without social engagement. The theory has achieved broad international recognition in both research and clinical practice [18,19], but it remains the subject of scientific debate, especially between Grossman [79], Neuhuber and Berthoud [80], and Porges [16–19].

The theory’s reception appears to be undermined by extremes. On the one hand, some practitioners treat it as a quasi-belief system, especially when they rely on anthropomorphic accounts of vagal states; on the other hand, some researchers default to biological reductionism. This polarization is partly intrinsic to the theory’s content. It happens when subjective concepts—like relationship quality—are treated as objectively based on neurophysiological structures or functioning. Phenomenological data are not mere “translations” of physiological markers. It is a category error to equate indicators (e.g., HRV, prosody) with meanings (e.g., safety, connectedness). From a phenomenological perspective, experience cannot be reduced to objective bodily measures, since meaning arises in lived experience (first person) and in second-person co-regulation [81,82]. Methodologically, this would imply triangulation of perspectives: (i) third-person measures such as HRV, respiratory patterns, acoustic prosody, and gait parameters; (ii) first-person assessments capturing bodily qualities (e.g., expansion–constriction); and (iii) second-person standardized observations of co-regulation (gaze, synchronization, vocal responsiveness) during group singing.

From our perspective, it would be useful to extend the theory’s bottom-up orientation by integrating top-down regulation—for example, by considering the coupling between freezing, perception, attention, and action preparation [83], and the influences of cortical control on autonomic regulation [84]. Especially, Julius Kuhl’s Theory of Personality Systems Interaction (PSI) [85,86] could frame autonomous regulation as “global affects” that constrain higher functions (e.g., self-access and volitional control) and shape lower-level functions (e.g., attentional shifting and intuitive behavior control). Complementing Polyvagal Theory with Kuhl’s Theory of Personality Systems Interaction (PSI) [7,8] could help differentiate how bottom-up autonomic states modulate top-down volition and motivation, yielding testable predictions—for example, that chronically defensive autonomic states might favor perseverative motor programs (e.g., festination, freezing of gait), whereas safety-inducing vocal and social cues would be expected to facilitate flexible action control and self-access.

Despite all limitations, we would like to point out that, epistemologically, theories are valuable not only when they invite verification or falsification. In the spirit of Karl Popper [87], theories can also serve as heuristic frameworks that guide discovery and stimulate the creative, integrative generation of testable hypotheses [18,19]. In conclusion, we therefore recommend not dismissing the theory prematurely but rather leveraging its innovative potential for Parkinson’s research. Beyond singing research, numerous PD findings—such as those on freezing of gait, apathy or facial masking—could be reconsidered in this light.

To highlight the potential of Polyvagal Theory for PD and singing, we designed the present study as a pilot study. The samples were heterogeneous, small, and selective. For example, the weekly singing group consisted predominantly of men, some of whom had no personal connection to singing in their biographies. During their usual training, we personally approached and motivated them to participate. This may have resulted in predominantly external motivation and contributed to dropouts and absenteeism. Furthermore, patients in both singing groups were rather active and joined health-related activities such as yoga, sports, and social events on their own accord. This may explain why the baseline values in body awareness were in the normal range and not significantly increased. On the one hand, these co-treatments may have created “noise” so that our present findings underestimate the effects of singing. On the other hand, the co-treatments may have helped participants to respond better to singing so that our present findings overestimate the effects of singing. Thus, the current pilot findings highlight the need for larger and more diverse samples in future studies.

Our patients reported that the body perception questionnaire addressed all of their specific symptoms of PD very well. This supports the face validity of our assumption that autonomic reactivity is important in studying PD. In addition, the patients reported differences in their symptoms during the on and off phases of medication. For example, patients reported increased respiratory rate as a typical feature during the off phases as opposed to the on phases. Future studies could assess and statistically control for the patients’ current phase. According to personal feedback, participants had some difficulties with the self-report of interoceptive sensibility. For example, it was not entirely clear to participants whether they had to evaluate the extent of their body discomfort (“To what extent do I have a dry mouth”) or their capacity for self-awareness and tracking one’s own body discomfort (“Do I really notice when having a dry mouth”). Future studies could solve this issue with additional instructions. To provide a more nuanced view, future studies could also rely on the full subscales of autonomic reactivity instead of the short proxy. The full subscales include body markers of a chronic dorsal vagal state as well as body markers of a chronic sympathetic state [51,56].

Future research should investigate whether patterns of autonomic reactivity are deciding factors in PD and which therapy can reverse these critical patterns. As a new approach to cope with PD, effects of singing could be compared to, for example, the evidence-based nervous system solutions *Safe and Sound Protocol,* informed by the Polyvagal Theory, as described on the Integrated Listening Systems website [88]. An alternative strategy for studying the effects of singing might be to focus on progression rather than assessing the regressive state of autonomic reactivity. Specifically, this approach would emphasize gathering information about the state of social engagement and related body markers. Recently, Morton and Cogan with their colleagues [89,90] published psychometric data supporting this strategy using the Neuroception of Psychological Safety Scale (NPSS), which contains markers of social engagement and safety.

Instead of the self-developed rating scale, we recommend: (i) use validated symptom instruments (e.g., quality of life, depression, anxiety), and (ii) instead of asking for broad physical/mental/emotional/social effects, capture phenomenological qualities such as expansion versus constriction known from trauma-or pain oriented procedures [91] and second-person standardized observations of co-regulation during group singing (gaze, synchronization, vocal responsiveness).

It is important to note that gaining insight into the symptom-oriented and subjectively lived reality of people with PD and its positive change, a multidimensional strategy is required that integrates embodiment with the socio-emotional context. Future studies should draw on more integrative theories and validate self-reports against non-reactive measures of autonomic reactivity—in particular 24-hour HRV assessment [92]—parallelized with videography of individual singing and group interactions.

## Conclusion

In the present pilot samples, we found preliminary, consistent results that align with the scope of *Singing Hospitals* to improve well-being through group singing. Specifically, we found that patients with PD reported greater bodily flexibility, enhanced mental clarity, deepened emotional resonance, and increased social connectedness immediately after group singing sessions. According to Polyvagal Theory, repeated activation of the social engagement system can, over time, foster dynamic regulation of autonomic states. This flexible physiological balance may lay the groundwork for coping and symptom mitigation. Our Hydra metaphor underscores PD’s “multi-headed” nature: managing one symptom may unmask or amplify others as motor and non-motor domains interact. Accordingly, strategies that target underlying regulatory dynamics are more likely to yield durable benefits. We therefore propose employing Polyvagal Theory as an innovative integrative lens to interpret the interwoven motor, non-motor, and psychosocial features of PD and to evaluate interventions. Our coping-formula is as follows: engage the social engagement system to support autonomic balance and counter symptom progression. Socially reinforced arts participation—especially group singing as conceptualized by Singing Hospitals—offers a promising, non-reductionist, low-threshold, and accessible format.

## Supporting information

S1 File Ethics application original. (PDF)

S2 File Ethics application English. (PDF)
