# Supplementary material for: Group singing through the lens of polyvagal theory: A pilot study in patients with Parkinson’s disease
Source: PLoS One. 2025 Dec 30;20(12):e0337210. doi: 10.1371/journal.pone.0337210 (PMC12752944; doi:10.1371/journal.pone.0337210)
Supplement: S1 File — (PDF) [file pone.0337210.s001.pdf]

Leitfaden
Antragstellung an die Ethik-Kommission der Universität Trier
(Stand 7/2003)
===

A Allgemeine Angaben

1. **Antragsteller**
   Prof. Dr. Nicola Baumann, Universität Trier, Fachbereich I

2. **Datum der Antragstellung**
   30.2.2019
   19. 12.2018 erfolgte bereits eine formal nicht korrekte Antragsstellung durch Elke Wünnenberg, die jetzt formal als Doktorandin angemeldet ist

3. **Titel des Forschungsvorhabens**
   Singen als Verhältnisprävention. Zugang zu Selbstregulation und Resilienzfaktoren

4. **Art der Studie**

   Single Case Experimental Study (Pilotstudie)

5. **Orte der Durchführung des Forschungsvorhabens**

   Pilotstudie erfolgt in Kooperation mit:
- *Sportwissenschaftlerin Dr. Mareike Schwed, neurowerkstatt Pfungstadt, https://www.neurowerkstatt.de/*
- *Fox Trial Finder, https://foxtrialfinder.michaeljfox.org/de/*

6. **Finanzierung**
   privat, Antrag auf Fördergelder werden über Ergebnisse der Vorstudien ggf. gestellt, Vorbereitung der Studie erfolgte über den Förderpreis der Hilde-Ulrichs-Stiftung für Parkinson-Forschung (10.000 Euro)

B Begründung des Forschungsvorhabens

1. Zusammenfassung

Menschen unterscheiden sich in ihrer Krisenkompetenz und in ihrem Umgang mit Erkrankungen. Alltagssprachlich werden diese Phänomene auch mit „Selbstheilungskräften“ beschrieben. Eigene klinische Erfahrungen, subjektive Erlebnisberichte und erste Forschungsergebnisse geben Hinweise darauf, dass über gemeinschaftliches, leistungsdruckfreies Singen ein Kontext geschaffen werden kann, in dem Selbstheilungskräfte wirksam werden und sich Krisenbewältigung verbessern lässt (Adamek 1996; Bossinger 2006; Wünnenberg, 2017). Die vorliegende Arbeit soll dieses Wirkpotenzial des Singens eingehender untersuchen und Grundlagen für weitere Interventionsstudien schaffen.

Es gibt eine Vielzahl möglicher Zugänge, um Selbstheilungskräfte zu erkunden. Auf der einen Seite existiert bislang ein globales Metakonzept der Resilienz, das personale, interpersonale Faktoren, Kontextfaktoren sowie Prozessfaktoren konfundiert (Rönnau-Böse &

Fröhlich-Gildhoff, 2015). Ebenso steht einer Fülle von Daten zur Herzratenvariabilität, als biologischer Marker für Resilienzkräfte ein mangelndes Verständnis zugrundeliegender Mechanismen gegenüber (Hamilton & Alloy, 2016). Auf der anderen Seite gibt es stark ausdifferenzierte Konstrukte (z.B. zur Selbstregulation und Affektregulation), die in unterschiedlichen Fachdisziplinen eher isoliert voneinander betrachtet werden.

Die hier gewählten Theorien der Persönlichkeits-System-Interaktionen (PSI) von Kuhl (2001) und der Polyvagaltheorie von Porges (1995, 2010, 2017) erlauben es, einige spezifische Zusammenhangsannahmen zwischen Stimmausdruck bzw. -wahrnehmung, vegetativer Dysfunktion, sozialer Bezogenheit, psychischem Befinden, Persönlichkeits- und Bewältigungsstilen und Motorik zu formulieren. Zentrale Grundannahme dieser Annahme ist, dass Singen Randbedingung schafft, die die vegetative Dysfunktion abmildern, psycho-vegetative Ressourcen zugänglich machen und so ein natürliches körperliches wie psychisches Bewegungsvermögen zurückzuholen vermag und körperliche wie psychische Erstarrungsphänomene abmildern kann (siehe Vagale Bremse nach Porges, 1995/2017; Modulationsannahmen nach Kuhl, 2001).

Die Pilotstudien sollen daher einerseits die Konstruktvalidität und Veränderungssensitivität der gewählten Forschungsmethoden wie z.B. Fragebögen zur Körperwahrnehmung (Porges, 1993; Cabrera et al., 2017), Herzratenvariabilitätsmessung (VNS-Analyse der Firma Commit), Entwicklungsorientierte Systemdiagnostik (Kuhl & Henseler, 2004), Resilienzfragebogen (Leppert et al., 2008) prüfen, andererseits über Einzelfallbetrachtungen und ihre Agglutination zu einer Single Case Experimental Study die Krisenkompetenzen von Parkinsonpatienten und das Wirkpotenzial des Singens zur Stärkung derselbigen verdeutlichen.

2. Forschungsstand

Nach 200 Jahren Parkinsonforschung gewinnen Strategien zur Prävention und Neuroprotektion (Fahn, 2018) einschließlich künstlerisch-aktivierender Verfahren wie Singen an Bedeutung. Integrative Ansätze zur Rehabilitation und/oder Prävention bei Parkinson zielen darauf ab, die Beeinträchtigungen zwischen dem Selbst als „Agens“ und seinem Körpersystem zu verringern und medikamentöse Behandlungsansätze zu erweitern. In einer Metaanalyse ordnen Uem et al. (2016) die eingehenden Artikel den unterschiedlichen Dimensionen der internationalen Klassifikation der Funktionsfähigkeit, Behinderung und Gesundheit (ICF) zu. Dabei konnten sie zeigen, dass die am ausführlichsten untersuchtesten Merkmale der Erkrankung nicht diejenigen sind, die am relevantesten für die Lebensqualität von Parkinsonpatienten sind. Insgesamt waren nicht-motorische Symptome enger mit einer reduzierten Lebensqualität der Patienten assoziiert als motorische Symptome. Garlovsky et al. (2016) identifizierte bis Dezember 2013 24 Studien, die nicht mehr biomedizinische, sondern psychische Faktoren für erhöhte Angstzustände und Depressionen untersuchten. Als signifikante Prädiktoren für diese psychische Symptomatik fanden sie verstärkte emotionsorientierte Bewältigung, weniger problemorientierte Bewältigung, niedrigere wahrgenommene Steuerung, Sicht auf Parkinson als Teil der Identität und großer Einflussfaktor auf das eigene Leben, geringere soziale Unterstützung und mehr vermeidende Persönlichkeitstypen. Dass gelingende Selbstregulation und positive Erwartungen zu Verbesserungen der Parkinsonsymptomatik führen können, deutet sich auch in der Placebo-Forschung an (Lidstone, 2014).

Im Zuge der Resilienzforschung zeigten Tugade und Frederiksen (2004) über einen multimethodischen Ansatz Zusammenhänge zwischen vegetativer Dysbalance, Affektregulation, Selbststeuerungsfähigkeiten und Resilienz auf: resiliente Menschen nutzen demnach positive Emotionen, um eine schnellere cardio-vaskuläre Erholung von negativer emotionaler Erregung zu erreichen und um positive Bedeutungszuschreibungen in negativen Umständen zu finden.

Kang, Scholp und Jiang (2017) berichten über die Veränderung der Aktivität neurologischer Netzwerke beim Singen, wie z.B. durch Ausschüttung von Oxytocin, Immunglobulin A und Endorphinen – hervorgerufen durch soziale Bindung oder durch Singen selbst –, die das Gefühl der sozialen Zugehörigkeit und damit gleichermaßen die Güte der Körperimmunfunktion verbessern und das persönliche Glückserleben verstärken. Die bisherigen Studienergebnisse zusammenfassend sprechen sie dem Singen ein therapeutisches Potenzial zu, weisen jedoch auf methodische Mängel (z.B. fehlende Kontrollgruppen) der Forschungsdesigns sowie auf noch unzureichendes Verständnis der zugrundeliegenden Wirkmechanismen hin.

Während es keine Singstudie für Patienten mit Posttraumatischer Belastungsstörung gibt, verwies Barnish (2016) aufgrund einer Metastudie auf einen möglichen therapeutischen Nutzen von Singen bei Parkinson. Während in der Parkinsonforschung erst begonnen wird, vegetative Dysbalance (Herzratenvariabilitäts-Parameter) als Erklärungsansatz für den Symptomverlauf heranzuziehen (Maetzler et al., 2009; Salsone, 2016; Shibata et al., 2009, In: Strano, Solla et al. 2015) ist dies in Bezug auf psychische Erkrankungen wie die posttraumatische Belastungsstörung zu einem wichtigen Indikator für den Krankheitsverlauf geworden. Zautra und seine Kollegen haben Resilienz als Vehikel für die Entstehung eines

neuen Gesundheitsmodells beschrieben (Reich, In: Drury, 2014). Dieses übt bereits eine vereinheitlichende Wirkung auf Beiträge aus dem synthetischen Feld der Positiven Psychologie und Evolutionspsychologie, der komplexen adaptiven Systemtheorie, der Genomik und der Neurowissenschaften aus. Insbesondere die Herzratenvariabilitätsmessung könnte künftig als psychobiologisches „Resilienzmaß“ neben anderen Biomarkern in Diagnose, Behandlung, Gesundheitsförderung, Krankheitsprävention und Leistungsoptimierung angewandt werden (Walkera et al., 2017) und wird von Hood, Begründer und Präsident des Institute for Systembiology (siehe unter www.systemsbiology.org) als 4P-Medizin (präventiv, personalisiert, präzise und partizipativ) propagiert. Hoffnung sei es, radikal individualisierte statt an einem „Goldstandard“ orientierte Behandlungsansätze zu entwickeln und die Kapazität und Fähigkeit zur Selbststeuerung in der Bevölkerung zu verbessern (Dury, 2014).

3. Ziel des Forschungsvorhabens

Gemäß den theoretischen Vorüberlegungen und der Augenscheinvalidität aus bisheriger Praxiserfahrungen soll erforscht werden, ob Singen einen Kontext schafft, in dem die Reduktion von Stresserleben und Abwehrverhalten, die Zunahme von innerer Sicherheit, Vitalität, Kontakterleben und damit eine Verbesserung von Selbstregulation und Abmilderung von Erstarrungsphänomenen möglich wird.
Es soll in mehreren Annäherungsschritten geprüft werden, inwiefern Singgruppenarbeit das psychische Funktionieren stärken und die Kapazität und Fähigkeit zu adaptiver Entwicklung unter der Randbedingung „Parkinson (PD)“ (ggf. im Vergleich mit Patienten einer „Posttraumatischer Belastungsstörung (PTBS)“) zu verbessern vermag. Desweiteren soll geprüft werden, inwiefern die beobachteten Veränderungen Porges/Kuhl folgen und in ein Wirkmodell überführt werden können, welche das etablierte, jedoch theoretisch wenig fundierte und ausdifferenzierte Resilienzkonzept genauern.

Die erhobenen Baseline-Daten, sollen nicht nur zur Beschreibung der Stichprobe und Hypothesentestung verwendet werden, sondern können auch zur Prüfung möglicher systematischer Stichprobenverzerrungen in der Pilotstudie herangezogen werden. Parallel zur Interventionsgruppe sollen nicht-singende Parkinsonpatienten (idealerweise Matched Pairs in Bezug auf Alter und/oder Krankheitsstand gemäß der Unified Parkinson's Disease Rating Scale UPDRS, Movement Disorder Society Task Force on Rating Scales for Parkinson's Disease, 2003; Freezing of Gait, FOG, Vogler et al., 2015) und ggf. Menschen mit posttraumatischen Belastungsstörungen anhand der Baseline-Daten der Entwicklungsorientierten Systemdiagnostik verglichen werden.

Entsprechend der Ergebnisse der Pilotstudie (Single Case Experimental Study) sollen nächste weitere Forschungsschritte (ggf. Hauptstudie) diskutiert und vorbereitet werden.

Folgende Forschungshypothesen (H1) sollen empirisch geprüft werden:

- Es gibt über alle Probanden hinweg negative Zusammenhänge zwischen der Kapazität zur Stressregulation (Vegetative Dysbalance) bzw. der Fähigkeit zur Selbststeuerung und der Parkinsonsymptomatik (insbesondere des körperlichen Freezings) und positive Zusammenhänge zwischen der Kapazität zur Stressregulation (Aktivität des Ventralen Vagus) bzw. Fähigkeit zur Selbststeuerung und Resilienz. Trifft es zu, dass die vegetative Dysfunktion der kritische Parameter ist, müssten die Daten des Körperfragebogens von Porges ebenso wie die Daten der Herzratenvariabilität die Stärke von Freezing-Phänomenen vorhersagen können.

- Die affektive Erstreaktion (d.h. die Affekt*sensibilität*, gemessen über Persönlichkeitsstile im PSSI oder stressreiche Lebensumstände im SSI) sowie unbewusste Motive und Motivumsetzung (Operanter Motivtest, OMT) haben über alle Probanden hinweg einen größeren Einfluss auf die Befindlichkeit/Symptomatik als die affektive Zweitreaktion (d.h. die Affekt*regulation*, gemessen über Selbststeuerungsfähigkeiten im SSI und bewusste Motive und Motivumsetzung im MUT).

- Symptome der Parkinsonerkrankten (u.a. motorische Einschränkungen) können in den Einzelfallstudien durch funktionsanalytische Betrachtungen nach Kuhl/Porges erklärt werden, indem sie mit einer horizontalen oder vertikalen Regression des individuellen psychischen Funktionierens (z.B. Selbst- oder Willlenshemmung) in Verbindung gebracht werden und sich beispielsweise in ängstlicher Selbstmotivierung oder fehlender Selbstberuhigungsfähigkeit zeigen. Psycho-vegetative Dysfunktion (Körperfragebogen, Herzratenvariabilität) begrenzt bzw. erweitert in den Einzelfallanalysen die Fähigkeit zur Selbststeuerung und spiegelt sich vor allem in einer prospektiven oder misserfolgsbezogenen Lage- bzw. Handlungsorientierung (im HAKEMP-Fragebogen) wider.

- Singen schafft einen Kontext, der kurzfristig zu einer Zunahme von Sicherheits- und Bezogenheitserleben und (körperlichem, emotionalem und geistigem) Wohlbefinden führt und langfristig zu einer verbesserten Selbststeuerung, einer Reduktion der Parkinsonsymptomatik und einer erhöhten Resilienz beiträgt.

- Implizite Motive beeinflussen stärker als explizite Motive das Wirkpotenzial des Singens. Menschen mit höheren Werten im impliziten Anschlussmotiv profitieren stärker vom Singen als Menschen mit einer Dominanz anderer Motive.

C Allgemeine Planung

1. Zusammenfassende Darstellung des Prüfungsablaufs

Aufgrund weniger Vorarbeiten wird das Projekt in einzelne Forschungsschritte unterteilt.

Nach Ermittlung des Forschungsstands, Wahl und Darstellung der theoretischen Annahmen und abgeleiteten Forschungshypothesen sollen Pilotstudien einerseits die Konstruktvalidität und Veränderungssensitivität der gewählten Forschungsmethoden prüfen, andererseits über Einzelfallbetrachtungen und ihre Agglutination zu einer Single Case Experimental Study die Krisenkompetenzen von Parkinsonpatienten und das Wirkpotenzial des Singens zur Stärkung derselbigen erforscht werden.

Pilotstudie

Mai 2019 Basline-Erhebung und Messung (prä) der Interventionsgruppe

	Baseline Erhebung über Fox-Trial-Finder der Kontrollgruppe (ohne Intervention, optional auch Patienten mit einer Posttraumatischen Störung ohne Parkinson oder andere neurologische Erkrankung)
Juni-Juli 2019	Intervention: 8 x 60min Singgruppe
Juli 2019	Messung (post)
November 2019	ggf. Katamnese

Vorbereitung Hauptstudie ab Herbst 2019

Geschätzter Zeitplan:

Winter 2018	Frühjahr 2019	Sommer 2019	Herbst 2019	Winter 2019	Frühjahr 2020	Sommer 2020
Literatur-studien	Pilot-studien	Pilotstudien		Haupt-studie	Hauptstudie	
Anträge	Ausarbeitung Theorie	Ausarbeitung ggf. Publikations-prozess	Ausarbeitung ggf. Publikations-prozess		Ausarbeitung ggf. Publikations-prozess	Ausarbeitung ggf. Publikations-prozess

2. Erhebungsmethoden und zum Einsatz kommende Verfahren

Überblick über geplante Verfahren:

Pilotphase

**Interventionen**	
Singen nach dem Konzept der Singenden Krankenhäuser e.V.	*www.singende-kranknhaeuser.de*<br>*Veröffentlichungen von E.Wünnenberg (2017)*
**Fragebogenerhebung**	
Anamnesebogen	Eigener Fragebogen zur Prüfung der Vorbehandlungen, Zugang zum Singen, s. Anhang
PSI-Funktionsanalyse der Persönlichkeit	https://impart.de/leistungen/testmodule/
Body Scales Questionnaire (BSQ)	http://stephenporges.com/index.php/publicationss/21-body-perception-questionnaires
United Parkinson Desaese Rating Scales UPDRS	http://www.neuromobil.ch/Uploads/Documents/0/UPDRS1.pdf
Freeze of Gait (FOG)	https://eprovide.mapi-trust.org/instruments/freezing-of-gait-questionnaire
Resilienzskala-RS 13	https://www.uniklinikum-jena.de/mpsy_media/Downloads/RESILIENZSKALA_LEPPERT2008.pdf
Visuelle Analogskala zur Befindlichkeit zu Beginn-Ender der Intervention	*Eigene Analogskala, s. Anhang*
**Psycho-physiologische Messung**	
Herzratenvariabilitätsmessung	https://www.vnsanalyse.de/

**Interventionen**	
Singen nach dem Konzept der Singenden Krankenhäuser e.V.	*www.singende-kranknhaeuser.de*<br>*Veröffentlichungen von E.Wünnenberg*
Optional:<br><br>Safe and Sound Protocol, Stephen Porges	*https://www.traumaheilung.net/SSP-Safe-and-Sound-Protocol.pdf*
Sensomotorischem Training & Stochastischem Resonanztraining (SRT)	
**Fragebögen**	
Body Scales Questionnaire (BSQ)	http://stephenporges.com/index.php/publicationss/21-body-perception-questionnaires
PSI-Funktionsanalyse der Persönlichkeit	https://impart.de/leistungen/testmodule/
Resilienzskala-RS 13/RS-25	https://www.uniklinikum-jena.de/mpsy_media/Downloads/RESILIENZSKALA_LEPPERT2008.pdf
Optional: Die Strukturale Analyse sozialen Verhaltens (Selbstumgang)	https://cip-medien.com/shop/sasb-die-strukturale-analyse-sozialen-verhaltens/
**Psycho-physiologische Messung**	
Herzratenvariabilitätsmessung, COMMIT	https://www.vnsanalyse.de/

1. **Geplanter Beginn und voraussichtliche Dauer**
   Frühjahr 2019 - Sommer 2020

In einem zweiten Forschungsschritt sollen (entsprechende Antragserweiterung wird der Ethikkommission im Verlauf der Pilotstudie nachgereicht) eine Interventionsstudie in Form einer Randomisierten Kontrollstudie (ggf. mit Wartegruppendesign), Multiple Baseline Design oder Randomisiertes Subjektdesign (Hauptstudie) erfolgen.

D Auswahl der Probandinnen/Probanden

1. Allgemeine Angaben

Patienten mit diagnostizierter Parkinsonerkrankung befinden sich in Einzel- und Gruppenangeboten die Stochastisches Resonanztraining (STR), Edukation und ein individuelles sportwissenschaftliches Trainingsprogramm an der Neurowerkstatt Pfungstadt absolvieren (mit unterschiedlicher Dauer und Frequenz). Alle bisherigen Therapien (Medikation, Physiotherapie werden dokumentiert und beibehalten. Die Rekrutierung erfolgt über Aushang und persönliche Ansprache über Dr. M. Schwed, Neurowerkstatt Pfungstadt.

Kontrollgruppe wird über Informationsschreiben an behandelnde Ärzte, Anschreiben von Selbsthilfegruppen und Fox Trial Finder sowie Annoncierung in der Zeitung erfolgen.

2. Ein- und Ausschlusskriterien für Probandinnen/Probanden

Einbindung in eine fachärztliche Behandlung zur Prüfung des Gesundheitszustands und Medikation

- Diagnose primäres Parkinson-Syndrom (G20.0-G20.2 nach ICD-10), ggf. mit komorbiden psychischen Folgestörungen, jedoch keiner weiteren neurologischen Erkrankung, Ausschluss von Demenz

- Zur Beschreibung der Stichprobe sowie Prüfung von Stichprobenverzerrungen werden zur Baseline-Erhebung ggf. Patienten mit der Diagnose „Reaktionen auf schwere Belastungen und Anpassungsstörungen" (F43.0-F43.2 nach ICD-10), ggf. mit komorbiden psychischen Folgestörungen, jedoch keine Andauernde Persönlichkeitsstörung (F 62) nach ICD-10 sowie Probanden ohne Diagnose einer körperlichen Erkrankung oder psychischen Störung einbezogen.

3. Ersetzung von Probandinnen/Probanden nach Aufnahme in das Forschungsvorhaben (im Falle vorzeitigen Abbruchs der Teilnahme)

Eine Ersetzung von Probandinnen/Probanden nach Aufnahme in das Forschungsprojekt ist nicht geplant.
Non-Responder und Dropout-Fälle werden dokumentiert.

4. Kontrolle des Gesundheitszustandes vor und während des Forschungsvorhabens

Die Prüfung des Gesundheitszustands erfolgt durch behandelnde Fachärzte der Probanden.

5. Abbruchkriterien

- Rückzug der Einverständniserklärung
- subjektiv wahrgenommene oder nach klinischen Kriterien diagnostizierte psychische oder körperliche Dekompensation

E Statistische Auswertung

Pilotstudien: Einzelfalldiagnostische Prüfung über Non-Overlap-Maße, nichtparametrische Tests, Randomisierungstests. Agglutination zu einer Single Case Experimental Study (Pospeschill & Siegel, 2018)

- Hauptstudie: Schätzung der Stichprobengröße nach MID Minimum Importent Difference, Lagemaße, Streuungsmaße, Zusammenhangsmaße, T-Test bzw. Kovarianzanalyse mit Baselinedaten

F Ethisch-rechtliche Aspekte

1. Bestehende Gesetze, Vorschriften, Richtlinien

Richtlinien des Fach- und Berufsverbands (Psychotherapeutenkammer BW)
Ethische Leitlinien der Initiative „Singende Krankenhäuser e.V.

2. Kosten-Nutzen-Abwägung

Aufgrund des schrittweisen Aufbaus (Pilotphase, Hauptstudie), den aus anerkannten wissenschaftlichen Theorien abgeleiteten Hypothesen und der Wahl valider Messinstrumente ist mit einem hohen Erkenntnisgewinn im Verhältnis zum eingesetzten personellen und finanziellen Aufwand zu rechnen.
Studien zu Verhältnisprävention bzw. Ansätze nach dem Konzept der Salutogenese/ Resilienz steigern Patientenkompetenzen und reduzieren Folgeerkrankungen, so dass die Studie den Forderungen der Steuerungsgruppe des Zukunftsforums Public Health (2017) entspricht.

3. Versicherungsschutz

Berufshaftpflichtversicherung der Versuchsleiterin, Gruppenhaftpflichtversicherung der zertifizierten Singleiter über Initiative Singende Krankenhäuser e.V. oder auftraggebende Einrichtungen der Singangebote

4. Vorprüfungen

Studie im Auftrag der Singenden Krankenhäuser e.V. von Kreutz, Böhm, Bossinger & Clift 2014/2015

5. Probandinnen/Probanden-Aufklärung/Einwilligungserklärung

Sehr geehrte Damen und Herren! Liebe Singbegeisterte und Singscheue!

Hiermit möchte ich Sie persönlich zur **Teilnahme an einer innovativen Studie zur Erforschung der heilsamen Wirkungen des Singens** im Umgang mit einer Parkinsonerkrankung einladen.
Die Studie wird im Januar 2019 beginnen und die Teilnahme an Singgruppen sowie begleitenden Interviews, Fragebogenbefragungen und innovativen psychovegetativen Behandlungsimpulsen enthalten.
Ziel der Studie ist aufzuzeigen, dass es Ansatzpunkte gibt, um einen Teufelskreis aus **körperlicher/emotionaler Erstarrung, Verlust von Selbstwirksamkeit, Kontakt und Vitalitätserleben** entgegenzuwirken. Statt Behandlung im klassischen Sinne geht es hier also um den Zugang und Erforschung von **individuellen Ressourcen**, die den Krankheitsverlauf

günstig beeinflussen, Defizite kompensieren und auf spielerische Weise Neuroprotektion und Gesundheitsprävention anregen.
Die Idee zu dieser Studie wurde von der Hilde-Ulrichs-Stiftung für Parkinsonforschung im September 2018 mit einem 10.000 Euro dotierten Förderpreis ausgezeichnet. Die Studie soll auch einen Beitrag leisten, um das Anliegen und Auftrag der gemeinnützigen internationalen Initiative **„Singende Krankenhäuser e.V.“** (siehe www.singende-krankenhaeuser.de) zu evaluieren und zu erweitern. Die Initiative setzt sich dafür ein, gesundheitsfördernde gemeinschaftliche Singangebote im Gesundheitssystem zu etablieren. Gesundheitseinrichtungen sollen damit zu einer Stätte der Begegnung werden, die die Einzigartigkeit, Würde und Beziehungsorientierung des Menschen als wichtigen Beitrag in der Behandlung aufzeigt. Ebenso erfolgt eine Koo https://www.angela-brantzen.de/peration mit der „neurowerkstatt“ Pfungstadt (Sportwissenschafterlin Dr. M. Schwed, www.neurowerkstatt.de) und Psychologin und zertifizierte Singleiterin Angela Brantzen (https://www.angela-brantzen.de/).

Die Studie findet im Rahmen einer Promotion unter Betreuung von Prof. Dr. Nicola Baumann statt und wurde von der Ethikkommission der Universität Trier genehmigt. Sie umfasst unterschiedliche Interventionen und Befragungen/Messungen, über diese nach einer zufälligen Zuordnung gesondert aufgeklärt werden. Kein Studienteilnehmer bekommt ein Placebo oder wird keiner Intervention unterzogen (außer ggf. Kontrollgruppe durch „gesunde Patienten“).
Ihre Teilnahme an der Studie ist freiwillig. Sie werden in diese Prüfung also nur dann einbezogen, wenn Sie dazu schriftlich Ihre Einwilligung erklären. Sofern Sie nicht an der Studie teilnehmen oder später aus ihr ausscheiden möchten, erwachsen Ihnen daraus keine Nachteile. Zu jedem Zeitpunkt können Sie ohne persönlichen Nachteil die Studie abbrechen oder die Verwendung Ihrer anonymisiert gespeicherten Daten zurückziehen. Mit der Teilnahme der Studie haben Sie ein Recht auf ein 30-50-minütiges persönliches Abschlussgespräch durch die Studienleiterin Elke Wünnenberg.
Der nachfolgende Text soll Ihnen die Ziele und den Ablauf erläutern. Anschließend wird ein Prüfarzt das Aufklärungsgespräch mit Ihnen führen. Bitte zögern Sie nicht, alle Punkte anzusprechen, die Ihnen unklar sind. Sie werden danach ausreichend Bedenkzeit erhalten, um über Ihre Teilnahme zu entscheiden.
Weitere Informationen entnehmen Sie bitte dem Anhang bzw. der Homepage www.singende-krankenhaeuser.de

Singen ist Medizin – was ist dran an dieser Aussage?

„Singen als Medizin“ ist zunächst einmal ein Slogan, um alle Beteiligten des Gesundheitssystems, insbesondere Patienten und ihre Behandler - auf das Potential des Singens aufmerksam zu machen. Der wichtigste Unterschied zu einer Arznei besteht darin, dass beim Singen die Wirkung nicht auf einer Substanz beruht, die von außen verabreicht wird. Singen spricht den Menschen in seinem Wesen an und aktiviert innere Selbstheilungskräfte. Noch dazu kann Singen zum spontanen Mitsingen anstecken – wirkt also vergleichbar wie ein „Gesundheitserreger.“

Spielt es für eine Rolle, was ich singe?

Entscheidender ist die Haltung, wie man singt. In dieser Studie werden einfache, lebensbejahende Lieder aller Kulturen gemeinschaftlich gesungen, die keinen Leistungsdruck zu erzeugen. Es sind keinerlei musikalische Vorkenntnisse notwendig.

Wie wirkt Singen konkret auf die Gesundheit?
Singen ist wie eine „Hotline“ zum autonomen Nervensystem und kann dieses ausbalancieren, insbesondere in Krisenzeiten, wenn chronische Stressreaktionen, unzureichende Bewältigungsstrategien und Rückzugsverhalten einen Teufelskreis erzeugen. Über ruhige, fliessende, besinnliche Lieder entlädt der Parasympathikus überschüssige Energie und entspannt den Körper, ermöglicht innere Versenkung und Flowerleben. Über schnellere strukturiert-rhythmische Lieder lädt der Sympathikus auf, energetisiert den Körper, werden Handlungsenergie, Mut und Tatkraft gestärkt.

Wie profitieren Parkinsonbetroffene davon?
Singen kann helfen, die Erfahrung zu machen, dass trotz Dopamindefizite Antrieb, Kontakt, Bewegungsfreude und Gemeinschaft möglich sind. Singen gibt spielerischen Zugang, was man/frau sonst oft über Training und Selbstdisziplin zu erreichen suchen. Musik hat ein Potenzial zur Selbstberuhigung und Selbstmotivierung – insbesondere im aktiven Singen. Der wichtigste Wirkfaktor ist, dass Singen über Atem und Klang körperliche wie emotionale Erstarrung lösen und Selbstwirksamkeit und Gemeinschaftserleben stärken kann.

Ist das wissenschaftlich belegt?
Einen Überblick über den wissenschaftlichen Erkenntnisstand bietet das Buch von Gunther Kreutz „Warum Singen glücklich macht“ sowie die Veröffentlichung der Initiative Singende Krankenhäuser e.V. „Singen als heilsame Kraft. Das Potenzial des Singens für das und Bindungsforschung wegweisend. Einem Überblicksartikel über sämtliche Studie über Singen mit Parkinsonpatienten von Barnish et al. 2018 wurden Hinweise auf ein therapeutisches Potenzial gefunden, die im Hinblick auf Kommunikation, kognitiven Status, Motorik und Lebensqualität noch genauer zu erforschen sind.

Studienleiterin und Ansprechpartnerin
Elke Wünnenberg (Psychologin, Psycholog. Psychotherapeutin, Dipl.-Musikerzieherin),

seit Gründung im Jahre 2009 Mitglied der Singenden Krankenhäuser e.V., seit 2012 dort im

Vorstand, seit 2016 Vorstandsvorsitzende. Preisträgerin der Hilde-Ulrichs-Stiftung für

Parkinsonforschung 2018.

Bitte um Kontaktaufnahme: elkewuennenberg@web.de; Tel: 0152-34531920

Mit meiner Unterschrift willige ich zur Kontaktaufnahme durch die Studienleiterin ein.

NAME	KONTAKTDATEN<br>(E-Mail, Telefon)	Unterschrift<br>(Einwilligung zur Kontaktaufnahme)

1. Vertraulichkeit von Probandinnen/Probandendaten

Datenspeicherung erfolgt in der Pilotphase pseudonymisiert über die Vergabe einer Patienten-ID, die nur der Studienleiterin zugänglich ist. Dabei werden die 2 ersten Anfangsbuchstaben des Vor- und Nachnamens über ein über Zufall generiertes Kodierungssystem (A wird zu I, B wird zu H....Z wird zu M) verschlüsselt. Dieser Kodierungsschlüssel wird verschlossen aufbewahrt und an keine Drittpersonen weitergegeben. Alle Fragebögen und Messungen sind mit dieser Kodierung und nicht mit dem Namen des Probanden versehen. Drittpersonen erhalten (Betreuerin, Kooperation Herzratenvariabilität, EOS-Testsystem) erhalten die Daten nur pseudonymisiert.

Die Daten werden auf einem privaten Buisness-Server/Private Cloud mit Backup-Funktion über Amazon) gespeichert und verschlüsselt. Zugriffsrecht hat nur durch die Studienleiterin. Nach Abschluss der Studie werde die Daten auf dem Server der Universität Trier, Fachbereich I, Abteilung Differentielle Psychologie, gespeichert. Die Probanden erhalten einen Ansprechpartner bei dem sie zu jedem Zeitpunkt die Löschung ihrer Daten beantragen können. Nach den Regeln zur Sicherung guter wissenschaftlicher Praxis werden die Daten generell auch bei Nichtverwendung erst nach 10 Jahren vernichtet.

Informed consent: Eine Einverständniserklärung zur Datenerhebung, Datenverarbeitung und Publikation auf Fachkonferenzen, Fachjournals wird nach einer Informationsveranstaltung bei den Probanden in der Datenschutzerklärung der Patienteneinwilligungserklärung schriftlich eingeholt. Am Ende der Studie erfolgt eine 30-50 persönliche Nachbesprechung durch die Studienleiterin mit jedem Probanden. Mit der Post-Messung wird eine schriftliche Einwilligung bei Probanden die ausdrückliche Erlaubnis eingeholt, die erhobenen Daten als Referenzdatum für künftige Studien der Studienleiterin weiterzuverwenden.
Nach den Regeln zur Sicherung guter wissenschaftlicher Praxis diese Daten erst nach 10 Jahren vernichtet werden.

2. Datenerfassung

Paper-Pencil :

- **Fragebogen zum Körpererleben (Porges)**
- **Resilienzfragebogen**

computergestützt

- System-Entwicklungsdiagnostik
- Herzratenvariabilitätsmessung über_VNS-Analyse, Commit GmbH www.commitgmbh.de

3. *Versicherung*

Der Abschluss einer verschuldensunabhängigen Probandenversicherung wird nicht für notwendig erachtet.

Verwendete Literatur:

Adamek, K. (1996). *Singen als Lebenshilfe. Zu Empirie und Theorie von Alltagsbewältigung*. Plädoyer für eine „Erneuerte Alltagskultur des Singens“. Münster: Waxmann.

Barnish J, Atkinson RA, Barran SM, Barnish MS. (2016).Potential Benefit of Singing for People with Parkinson's Disease: A Systematic Review. J Parkinsons Dis. 3;6(3):473-84. doi: 10.3233/JPD-160837.

Blechert, J., Michael, T., Grossman, P., Lajtman, M., & Wilhelm, F. H. (2007). Autonomic and respiratory characteristics of posttraumatic stress disorder and panic disorder. *Psychosomatic Medicine*, *69*(9), 935–943. https://doi.org/10.1097/PSY.0b013e31815a8f6b

Bossinger, W. (2006). *Die heilende Kraft des Singens. Von den Ursprüngen bis zu modernen Erkenntnissen über die soziale und gesundheitsfördernde Wirkung von Gesang*. Battweiler: Traumzeit.

Briegel, W., Walter, T., Schimek, M., Knapp, D., & Bussing, R. (2015). Parent-Child Interaction Therapy im In-room-Coaching. Ergebnisse einer ersten deutschen Fallstudie. *Kindheit Und Entwicklung*, *24*(1), 47–54. https://doi.org/10.1026/0942-5403/a000158

Cho, K.-H., Kim, T.-H., Kwon, S., Jung, W.-S., Moon, S.-K., Ko, C.-N., … Chung, E. K. (2018). Complementary and alternative medicine for idiopathic Parkinson’s disease: An evidence-based clinical practice guideline. *Frontiers in Aging Neuroscience*, *10*. https://doi.org/10.3389/fnagi.2018.00323

Combs HL, Garcia-Willingham NE, Berry DTR, van Horne CG, Segerstrom SC. (2018). Psychological functioning in Parkinson's disease post-deep brain stimulation: Self-regulation and executive functioning. J Psychosom Res.;111:42-49. doi: 10.1016/j.jpsychores.2018.05.007. Epub 2018 May 21.

Christian T. Haas, Stephan Turbanski, Dietmar Schmidtbleicher (2006).Wie gezielte Unordnung im Training für Ordnung in der Bewegung sorgt Zufällige Schwingungen wirken auf Muskel- und Nervenzellen ein. Sensomotorischem Training & Stochastischem Resonanztraining (SRT). Forschungs Frankfurt 4, 19-25.

Cossu, G., Rinaldi, R., & Colosimo, C. (2018). The rise and fall of impulse control behavior disorders. *Parkinsonism & Related Disorders*, *46*(Suppl 1), S24–S29. https://doi.org/10.1016/j.parkreldis.2017.07.030

Dale, L. P., Shaikh, S. K., Fasciano, L. C., Watorek, V. D., Heilman, K. J., & Porges, S. W. (2017). College Females With Maltreatment Histories Have Atypical Autonomic Regulation and Poor Psychological Wellbeing. *Psychological Trauma: Theory, Research, Practice, andPolicy*. Advance online publication. http://dx.doi.org/10.1037/tra0000342

De Bock, F., Geene, R., Hoffmann, W., Stang, A. (2017): Handreichung aus der Steuerungsgruppe des Zukunftsforums Public Health für alle mit Prävention in Praxis und Politik befassten Akteure Vorgelegt von einer Ad-hoc-AG durch Zukunftsforum Public Health, Berlin, Verfügbar unter: https://zukunftsforum-public-health.de/wp-content/uploads/2018/01/2017_12_Handreichung_Verh%C3%A4ltnispr%C3%A4vention_Zukunftsforum.pdf

Devereaux (2017) An Interview with Dr. Stephen W. Porges- Am J Dance Ther 39:27–35. DOI 10.1007/s10465-017-9252-6

Drury, R. (2014). Wearable biosensor systems and resilience: a perfect storm in health care? Review Article. *Frontiers in Psychology*, 5, 853, 1-5. DOI: 10.3389/fpsyg.2014.00853

Faherty, C. J., Shepherd, K. R., Herasimtschuk, A., & Smeyne, R. J. (2005). Environmental enrichment in adulthood eliminates neuronal death in experimental Parkinsonism. *Molecular Brain Research*, *134*(1), 170–179. https://doi.org

Fahn, S. (2018). The 200-year journey of Parkinson disease: Reflecting on the past and looking towards the future. Parkinsonism and Related Disorders 46 (2018) S1eS

Faul, F., Erdfelder, E., Lang, A.-G., & Buchner, A. (2007). G*Power 3: A flexible statistical power analysis program for the social, behavioral, and biomedical sciences. Behavior Research Methods, 39, 175-191. Download PDF

Green KT1, Dennis PA2, Neal LC3, Hobkirk AL4, Hicks TA4, Watkins LL4, Hayano J5, Sherwood A4, Calhoun PS6, Beckham JC6. (2016). Exploring the relationship between posttraumatic stress disorder symptoms and momentary heart rate variability. J Psychosom Res. 2016 Mar;82:31-4. doi: 10.1016/j.jpsychores.2016.01.003. Epub 2016 Jan 13.

Flores Alves Dos Santos, J., Tezenas du Montcel, S., Gargiulo, M., Behar, C., Montel, S., Hergueta, T., … Welter, M.-L. (2017). Tackling psychosocial maladjustment in Parkinson's disease patients following subthalamic deep-brain stimulation: A randomised clinical trial. *PLoS ONE*, *12*(4).

Garlovsky, J. K., Overton, P. G., & Simpson, J. (2016). Psychological predictors of anxiety and depression in Parkinson's disease: A systematic review. *Journal of Clinical Psychology*, *72*(10), 979–998. https://doi.org/10.1002/jclp.22308

Hamilton, J. & Alloy, L. (2016): Atypical reactivity of heart rate variability to stress and depression across development: Systematic review of the literature and directions for future research. *Clinical Psychology Review,* 50, 67–79. DOI: 10.1016/j.cpr.2016.09.003. Epub 2016 Sep 20

Hautzinger M. (1994). Action control in the context of psychopathological disorders. In J. Kuhl & J.Beckmann (Hrsg.), Volition and personality: Action versus state orientation (S.209-215). Seattle: Hogrefe.

Hawkes, C.H., Del Tredici, K., Braak, H. (2010). A timeline for Parkinson's disease. Parkinsonism and Related Disorders 16 (2010) 79–84

Heller, L. & Lapierre, A.(2013). *Entwicklungstrauma heilen. Alte Überlebensstrategien lösen – Selbstregulierung und Beziehungsfähigkeit stärken – Das Neuroaffektive Beziehungsmodell zur Traumaheilung NARM.* München: Kösel.

Holt-Lunstad, J., Smith, T. & Layton, J. (2010). Social Relationships and Mortality Risk: A Meta-analytic Review. *PLoS Medicine, 7* (7). https://doi.org/10.1371/journal.pmed.1000316.

Holzman JB, Bridgett DJ. Heart rate variability indices as bio-markers of top-down self-regulatory mechanisms: A meta-analytic review. Neurosci Biobehav Rev. 2017 Mar;74(Pt A):233-255. doi: 10.1016/j.neubiorev.2016.12.032. Epub 2017 Jan 3.

Jungen, M. Persönliche Gespräche und Unveröffentlichte Unterlagen aus Resilienztrainings und Projekten, in denen organisationale Resilienzkonzepte realisiert wurden.

Kang, J & Scholp, A. & Jiang, J. (2017). A Review of the Physiological Effects and Mechanisms of Singing. *Journal of Voice*. DOI: 10.1016/j.jvoice.2017.07.008.

Kempa, A.H, Julian Koenigc, Julian F. Thayerd (2017). From psychological moments to mortality: A multidisciplinary synthesis on heart rate variability spanning the continuum of time. Neuroscience and Biobehavioral Reviews 83, 547–567

Klaissle, P., Lesemann, A., Huehnchen, P., Hermann, A., Storch, A., & Steiner, B. (2012). Physical activity and environmental enrichment regulate the generation of neural precursors in the adult mouse substantia nigra in a dopamine-dependent manner. *BMC Neuroscience*, *13*. Retrieved from http://www.redi-bw.de/db/ebsco.php/search.ebscohost.com/login.aspx%3fdirect%3dtrue%26db%3dpsyh%26AN%3d2014-50040-001%26site%3dehost-live

Kreutz, G. (2014). *Warum Singen glücklich macht*. Gießen: Psychosozial-Verlag.

Kreutz, G., Clift, S., Böhm, K. & Bossinger, W. (2017). *Singende Krankenhäuser aus Sicht von Singgruppenleiter/innen.* In E. Wünnenberg (Hrsg.), Singen als heilsame Kraft. Das Potenzial des Singens für das Gesundheitssystem. Grundlagen-Praxisfelder-Perspektiven, 2. erw. Aufl., 164-190, Bad Waldsee: Selbstverlag.

Kuhl, J. (2001). *Motivation und Persönlichkeit. Interaktion psychischer Systeme.* Göttingen: Hogrefe.

Lenka, A., Hegde, S., Arumugham, S. S., & Pal, P. K. (2017). Pattern of cognitive impairment in patients with Parkinson's disease and psychosis: A critical review. *Parkinsonism & Related Disorders*, *37*, 11–18. https://doi.org/10.1016/j.parkreldis.2016.12.025

Leppert, Koch, Bräher & Strauß (2008). Die Resilienzskala. Überprüfung der Langform RS-25 und einer Kurzform RS-13. Klin. Diagnostik und Evaluation, 1, 226-243

Lidstone SC (2014). Great expectations: the placebo effect in Parkinson's disease. Handb Exp Pharmacol. 2014;225:139-47. doi: 10.1007/978-3-662-44519-8_8.

Maetzler W1, Liepelt I, Berg D. (2009). Progression of Parkinson's disease in the clinical phase: potential markers. Lancet Neurol. 2009 Dec;8(12):1158-71. doi: 10.1016/S1474-4422(09)70291-1.

Macht, M., & Ellgring, H. (1999). Behavioral analysis of the freezing phenomenon in Parkinson's disease: a case study. *Journal of Behavior Therapy and Experimental Psychiatry*, *30*, 241–247. https://doi.org/10.1016/S0005-7916(99)00021-X

Marlysa B. Sullivan, Matt Erb, Laura Schmalzl, Steffany Moonaz,

Marques, A., Durif, F., & Fernagut, P.-O. (2018). Impulse control disorders in Parkinson's disease. *Journal of Neural Transmission*, *125*(8), 1299–1312. https://doi.org/10.1007/s00702-018-1870-8

Muehsam. D. Lutgendorf, S., Mills, P.J., Rickhi, B., Chevalier, G., Bat, N. , Chopra, D., Gurfein, B. (2017). The embodied mind: A review on functional genomic and neurological correlates of mind-body therapies. Neuroscience & Biobehavioral Reviews, 73, 165-181. https://doi.org/10.1016/j.neubiorev.2016.12.027

Nithianantharajah, J., & Hannan, A. J. (2006). Enriched environments, experience-dependent plasticity and disorders of the nervous system. *Nature Reviews Neuroscience*, *7*(9), 697–709. https://doi.org/10.1038/nrn1970

Park, J. E., Lee, J. Y., Kang, S.-H., Choi, J. H., Kim, T. Y., So, H. S., & Yoon, I.-Y. (2017). Heart rate variability of chronic posttraumatic stress disorder in the Korean veterans. *Psychiatry Research*, *255*, 72–77. https://doi.org/10.1016/j.psychres.2017.05.011

Petkus, Andrew J., J. Vincent Filoteo, Green, K. T., Dennis, P. A., Neal, L. C., Hobkirk, A. L., Hicks, T. A., Watkins, L. L., Beckham, J. C. (2016). Exploring the relationship between posttraumatic stress disorder symptoms and momentary heart rate variability. *Journal of Psychosomatic Research*, *82*, 31–34. https://doi.org/10.1016/j.jpsychores.2016.01.003

Porges S.W. (1995). Orienting in a defensive world: mammalian modifications of our evolutionary heritage. A Polyvagal Theory. Psychophysiology. 32(4):301-18.

Porges, S.W. (2010). *Die Polyvagal-Theorie: Neurophysiologische Grundlagen der Therapie*. Paderborn: Junfermann.

Porges, S.W. (2017). *Die Polyvagaltheorie und die Suche nach Sicherheit. Traumabehandlung, soziales Engagement und Bindung*. Gespräche und Reflexionen zur Polyvagaltheorie. Lichtenau: Probst.

Porges, S.W. (2007). A phylogenetic journey through the vague and ambiguous Xth cranial nerve: a commentary on contemporary heart rate variability research. Biol Psychol.;74(2):301-7. DOI:10.1016/j.biopsycho.2006.08.007

Pospeschill, M. & Siegel, R. (2018) Methoden für die klinische Forschung und diagnostische Praxis. Heidelberg: Springer-Verlag.

Rönnau-Böse M. (2014) Resilienz. "3., aktualisierte Auflage." München, Basel: Ernst Reinhardt.

Rönnau-Böse, M., & Fröhlich-Gildhoff, K. (2015). Resilienz und Resilienzförderung über die Lebensspanne. Kohlhammer Verlag

Rüegg (2001): Psychosomatik, Psychotherapie und Gehirn. Neuronale Plastizität als Grundlage einer biopsychosozialen Medizin. Stuttgart: Schattauer.

Salsone M, Vescio B, Fratto A, Sturniolo M, Arabia G, Gambardella A, Quattrone A. (2016). Cardiac sympathetic index identifies patients with Parkinson's disease and REM behavior disorder.Parkinsonism Relat Disord. 26:62-6. doi: 10.1016/j.parkreldis.2016.03.004. Epub 2016 Mar 10.

Schiavio, A. & Altenmüller, E. (2015): Exploring music-based rehabilitation for Parkinsonism through embodied cognitive science. Frontiers in Neurology. https://doi.org/10.3389/fneur.2015.00217

Schiepek, G. & Matschi, B. (2013). Ressourcenerfassung im therapeutischen Prozess. Darstellung, Förderung und nachhaltige Nutzung. Psychotherapie im Dialog, 14,1, 56-61. DOI: 10.1055/s-0033-1337098

Seligowski, A. V., Lee, D. J., Bardeen, J. R., & Orcutt, H. K. (2015). Emotion regulation and posttraumatic stress symptoms: A meta-analysis. *Cognitive Behaviour Therapy*, *44*(2), 87–102. https://doi.org/10.1080/16506073.2014.980753

Sullivan, M.,Erb, M. Schmalzl, L.,Moonaz, S.Noggle, J. Taylor & Porges, S.W.(2018).The Convergence of Traditional Wisdom and Contemporary Neuroscience for Self-Regulation and Resilience, Front. Hum. Neurosci. https://doi.org/10.3389/fnhum.2018.00067

Strano S, Fanciulli A, Rizzo M, Marinelli P, Palange P, Tiple D, De Vincentis G, Calcagnini G, Censi F, Meco G, Colosimo C. (2016). Cardiovascular dysfunction in untreated Parkinson's disease: A multi-modality assessment. J Neurol Sci.;370:251-255. doi: 10.1016/j.jns.2016.09.036. Epub 2016 Sep 21.

Solla P1, Cadeddu C2, Cannas A1, Deidda M3, Mura N3, Mercuro G3, Marrosu F1.(2015). Heart rate variability shows different cardiovascular modulation in Parkinson's disease patients with tremor dominant subtype compared to those with akinetic rigid dominant subtype. J Neural Transm (Vienna).122(10):1441-6. doi: 10.1007/s00702-015-1393-5. Epub 2015 Mar 24.

Tass, P. A., Qin, L., Hauptmann, C., Dovero, S., Bezard, E., Boraud, T., & Meissner, W. G. (2012). Coordinated reset has sustained aftereffects in Parkinsonian monkeys. *Annals of Neurology*, *72*(5), 816–820. https://doi.org/10.1002/ana.23663

Thiriet, N., Amar, L., Toussay, X., Lardeux, V., Ladenheim, B., Becker, K. G., … Jaber, M. (2008). Environmental enrichment during adolescence regulates gene expression in the striatum of mice. *Brain Research*, *1222*, 31–41. https://doi.org/10.1016/j.brainres.2008.05.030

Trojano, L., & Papagno, C. (2018). Cognitive and behavioral disorders in Parkinson’s disease: An update II: Behavioral disorders. *Neurological Sciences*, *39*(1), 53–61. https://doi.org/10.1007/s10072-017-3155-7

Trösken A.K. (2010). Das Berner Ressourceninventar. Ressourcenpotentiale und Ressourcenrealisierung aus konsistenztheoretischer Sicht Inauguraldissertation der Philosophisch-historischen Fakultät der Universität Bern zur Erlangung der Doktorwürde vorgelegt von Bundesrepublik Deutschland Institut für Psychologie Universität Bern

Tugade, M. M., & Fredrickson, B. L. (2004). Resilient Individuals Use Positive Emotions to Bounce Back From Negative Emotional Experiences. *Journal of Personality and Social Psychology*, *86*(2), 320–333. https://doi.org/10.1037/0022-3514.86.2.320

Uem, J. M. T., Marinus, J., Canning, C., van Lummel, R., Dodel, R., Liepelt-Scarfone, I., … Maetzler, W. (2016). Health-Related Quality of Life in patients with Parkinson’s disease—A systematic review based on the ICF model. *Neuroscience and Biobehavioral Reviews*, *61*, 26–34. https://doi.org/10.1016/j.neubiorev.2015.11.014

Walkera, F., Pfingst, K., Carnevalic, L., Sgoifoc, A. & Nalivaikoa, E. (2017). In the search for integrative biomarker of resilience to psychological stress, *Neuroscience and Biobehavioral Reviews*, 74, 310–320, DOI: 10.1016/j.neubiorev.2016.05.003. Epub 2016 May 11

Wünnenberg, E. (2017a). *Singend bezogen sein. Entwurf einer Theorie & Methodik des heilsamen Singens zur Krankheitsbewältigung und Gesundheitsfürsorge,* In E. Wünnenberg (Hrsg.), Singen als heilsame Kraft. Das Potenzial des Singens für das Gesundheitssystem. Grundlagen-Praxisfelder-Perspektiven, 2. erw. Aufl., 76-116, Bad Waldsee: Selbstverlag.

Wünnenberg, E. (2017b). *Singen & Resilienz. Vom Singen zum Sein. Wie singen uns stärkt: Selbstregulation auf tönende Weise,* In E. Wünnenberg (Hrsg.), Singen als heilsame Kraft. Das Potenzial des Singens für das Gesundheitssystem. Grundlagen-Praxisfelder-Perspektiven, 2. erw. Aufl., 118-148, Bad Waldsee: Selbstverlag.

Wünnenberg (erscheint im Frühjahr 2019). Singende Krankenhäuser e.V. In; Decker-Voigt, H. & Weymann (Hrgs): Musiktherapie-Lexikon. Göttingen: Hogrefe.

Wünnenberg (erscheint im Frühjahr 2019). Selbstregulation – adaptive Entwicklung - Krisenkompetenzen. In; Decker-Voigt, H. & Weymann (Hrgs): Musiktherapie-Lexikon. Göttingen: Hogrefe.

Xian-Si Zeng*, Wen-Shuo Geng, Jin-Jing Jia*, Lei Chen and Peng-Peng Zhang (2018).Cellular and Molecular Basis of Neurodegeneration in Parkinson Disease, doi: 10.3389/fnagi.2018.00109

Xie, C.-L., Wang, X.-D., Chen, J., Lin, H.-Z., Chen, Y.-H., Pan, J.-L., & Wang, W.-W. (2015). A systematic review and meta-analysis of cognitive behavioral and psychodynamic therapy for depression in Parkinson’s disease patients. *Neurological Sciences*, *36*(6), 833–843. https://doi.org/10.1007/s10072-015-2118-0
