# Supplementary material for: Group singing through the lens of polyvagal theory: A pilot study in patients with Parkinson’s disease
Source: PLoS One. 2025 Dec 30;20(12):e0337210. doi: 10.1371/journal.pone.0337210 (PMC12752944; doi:10.1371/journal.pone.0337210)
Supplement: S2 File — (PDF) [file pone.0337210.s002.pdf]

Guide
Application to the Ethics Committee of the University of Trier (as of 7/2003)
==

A General information

1. Applicant
   Prof. Dr. Nicola Baumann, University of Trier, Faculty I

2. Date of application
   30.2.2019
   19. 12.2018 Elke Wünnenberg, who is now formally registered as a doctoral student, has already submitted an application that is not formally correct

3. Title of the research project
   Singing as Relationship Prevention. Access to self-regulation and resilience factors

4. Type of study

   Single Case Experimental Study (Pilotstudie)

5. Pilot study is carried out in cooperation with:
- *Sports scientist Dr. Mareike Schwed, neurowerkstatt Pfungstadt, https://www.neurowerkstatt.de/*
- *Fox Trial Finder, https://foxtrialfinder.michaeljfox.org/de/*

6. Financing
   private, application for funding may be made on the basis of the results of the preliminary studies, preparation of the study was carried out via the Hilde Ulrichs Foundation Award for Parkinson's Research (10,000 euros)

B Rationale for the research project

1. Summary

People differ in their crisis competence and in how they deal with illnesses. In everyday language, these phenomena are also described as "self-healing powers". Own clinical experiences, subjective reports of experiences and initial research results indicate that a context can be created through communal, performance-free singing in which self-healing powers become effective and crisis management can be improved (Adamek 1996; Bossinger 2006; Wünnenberg, 2017). The present work aims to investigate this potential effect of singing in more detail and to create a basis for further intervention studies.

There are a variety of possible approaches to explore self-healing powers. On the one hand, there is a global meta-concept of resilience that confuses personal, interpersonal factors, contextual factors and process factors (Rönnau-Böse & Fröhlich-Gildhoff, 2015). Similarly , a wealth of data on heart rate variability, as a biological marker of resilience forces, is offset by a lack of understanding of underlying mechanisms (Hamilton & Alloy, 2016). On the other hand, there are highly differentiated constructs (e.g. on self-regulation and affect regulation) that are considered in isolation from each other in different disciplines.

The theories of personality-system interactions (PSI) of Kuhl (2001) and the polyvagal theory of Porges (1995, 2010, 2017) chosen here allow us to formulate some specific assumptions between voice expression and perception, vegetative dysfunction, social relatedness, psychological well-being, personality and coping styles, and motor skills. The central basic assumption of this assumption is that singing creates boundary conditions that mitigate vegetative dysfunction, make psycho-vegetative resources accessible and thus can regain a natural physical and psychological ability to move and mitigate physical and psychological paralysis phenomena (see Vagale Bremse according to Porges, 1995/2017; Modulation assumptions according to Kuhl, 2001).

The pilot studies are therefore intended to assess the construct validity and change sensitivity of the chosen research methods, such as questionnaires on body perception (Porges, 1993; Cabrera et al., 2017), heart rate variability measurement (VNS analysis of the company Commit), development-oriented system diagnostics (Kuhl & Henseler, 2004), resilience questionnaire (Leppert et al., 2008), on the other hand to illustrate the crisis competencies of Parkinson's patients and the effective potential of singing to strengthen them by means of individual case considerations and their agglutination to a single case experimental study.

2. State of research

After 200 years of Parkinson's research, strategies for prevention and neuroprotection (Fahn, 2018), including artistically activating procedures such as singing, are gaining in importance. Integrative approaches to rehabilitation and/or prevention in Parkinson's aim to reduce the impairments between the self as an "agent" and its body system and to expand drug treatment approaches. In a meta-analysis, Uem et al. (2016) assign the incoming articles to the different dimensions of the International Classification of Functioning, Disability and Health (ICF). They were able to show that the most extensively studied characteristics of the disease are not those that are most relevant to the quality of life of Parkinson's patients. Overall, non-motor symptoms were more closely associated with reduced quality of life in patients than motor symptoms. Garlovsky et al. (2016) identified 24 studies as of December 2013 that no longer investigated biomedical, but psychological factors for increased anxiety and depression. As significant predictors of this psychological symptomatology, they found increased emotion-oriented coping, less problem-oriented coping, lower perceived control, view of Parkinson's as part of one's identity and major influencing factor on one's own life, lower social support and more avoidant personality types. The fact that successful self-regulation and positive expectations can lead to improvements in Parkinson's symptoms is also indicated in placebo research (Lidstone, 2014).

In the course of resilience research, Tugade and Frederiksen (2004) used a multimethodological approach to show connections between vegetative imbalance, affect regulation, self-control skills and resilience: resilient people use positive emotions to achieve faster cardio-vascular recovery from negative emotional arousal and to find positive attributions of meaning in negative circumstances.

Kang, Scholp and Jiang (2017) report changes in the activity of neurological networks during singing, such as through the release of oxytocin, immunoglobulin A and endorphins – caused by social bonding or by singing itself – which improve the sense of social belonging and thus equally the goodness of the body's immune function and enhance the personal experience of happiness. Summarizing the results of the study so far, they attribute a therapeutic potential to singing, but point to methodological deficiencies (e.g. lack of control groups) in the research designs as well as to insufficient understanding of the underlying mechanisms of action.

While there is no singing study for patients with post-traumatic stress disorder, Barnish (2016) referred to a possible therapeutic benefit of singing in Parkinson's disease based on a meta-study. While Parkinson's research is only beginning to use vegetative imbalance (heart rate variability parameters) as an explanatory approach for the course of symptoms (Maetzler et al., 2009; Salsone, 2016; Shibata et al., 2009, In: Strano, Solla et al. 2015), this has become an important indicator of the course of the disease in relation to mental illnesses such as post-traumatic stress disorder. Zautra and his colleagues have identified resilience as a vehicle for the emergence of a new health model (Reich, In: Drury, 2014). This already has a unifying effect on contributions from the synthetic field of positive psychology and evolutionary psychology, complex adaptive systems theory, genomics and neuroscience. In particular, heart rate variability measurement could be used in the future as a psychobiological "resilience measure" alongside other biomarkers in diagnosis, treatment, health promotion, disease prevention, and performance optimization (Walkera et al., 2017) and is proposed by Hood, founder and president of the Institute for Systems Biology (see under www.systemsbiology.org) as 4P medicine (preventive, personalized, precise and participatory). The hope is to develop radically individualized treatment approaches instead of a "gold standard" and to improve the capacity and ability for self-control in the population (Dury, 2014).

3. Aim of the research project

According to the theoretical preliminary considerations and the apparent validity from previous practical experience, it is to be investigated whether singing creates a context in which the reduction of stress experience and defensive behavior, the increase of inner security, vitality, contact experience and thus an improvement of self-regulation and mitigation of numbness phenomena becomes possible.
In several approach steps, it will be examined to what extent singing group work strengthens psychological functioning and increases the capacity and ability for adaptive development under the boundary condition "Parkinson's disease (PD)" (possibly Parkinson's disease). compared with patients with a
"Post-traumatic stress disorder (PTSD)"). Furthermore, it will be examined to what extent the observed changes follow Porges/Kuhl and can be transferred into an effective model that more precisely reflects the established, but theoretically unfounded and differentiated concept of resilience.

The collected baseline data will not only be used to describe the sample and hypothesis testing, but can also be used to test possible systematic sampling biases in the pilot study. Parallel to the intervention group, non-singing Parkinson's patients (ideally matched pairs in terms of age and/or disease status according to the Unified Parkinson's Disease Rating Scale UPDRS, Movement Disorder Society Task Force on Rating Scales for Parkinson's Disease, 2003; Freezing of Gait, FOG, Vogler et al., 2015) and, if necessary, people with post-traumatic stress disorder were compared using baseline data from development-oriented systems diagnostics.

According to the results of the pilot study (Single Case Experimental Study), the next further research steps (possibly main study) are to be discussed and prepared.

The following research hypotheses (H1) are to be empirically tested:

- There are negative associations across all subjects between the capacity for stress regulation (vegetative imbalance) or the ability to self-control and Parkinson's symptoms (especially physical freezing) and positive associations between the capacity for stress regulation (activity of the ventral vagus) or the ability for self-control and resilience. If it is

true that vegetative dysfunction is the critical parameter, the data from the Porges body questionnaire should be able to predict the severity of freezing phenomena, as should the data on heart rate variability.

- The affective first reaction (i.e. the sensitivity of affect, measured by personality styles in the PSI or stressful living conditions in the SSI) as well as unconscious motives and motive implementation (operant motive test, OMT) have a greater influence on the state of mind/symptomatology than the affective second reaction (i.e. the affect regulation, measured by self-control skills in the SSI and conscious motives and motive implementation in the MUT) across all subjects.

- Symptoms of Parkinson's patients (including motor impairments) can be explained in individual case studies by functional analysis according to Kuhl/Porges, in that they are associated with a horizontal or vertical regression of individual psychological functioning (e.g. self-inhibition or inhibition of will) and manifest themselves, for example, in anxious self-motivation or lack of self-calming ability. Psycho-vegetative dysfunction (body questionnaire, heart rate variability) limits or expands the ability to self-control in the individual case analyses and is reflected above all in a prospective or failure-related situation or action orientation (in the HAEMP questionnaire).

- Singing creates a context that leads to an increase in the experience of safety and relatedness and (physical, emotional and mental) well-being in the short term and contributes to improved self-control, a reduction in Parkinson's symptoms and increased resilience in the long term.

- Implicit motifs influence the potential of singing more strongly than explicit motifs. People with higher values in the implicit follow-up motive benefit more from singing than people with a dominance of other motives.

C General Planning

1. Summary of the examination procedure

Due to less preparatory work, the project will be divided into individual research steps.

After determining the state of research, selection and presentation of the theoretical assumptions and derived research hypotheses, pilot studies will test the construct validity and change sensitivity of the chosen research methods on the one hand, and on the other hand the crisis competencies of Parkinson's patients and the effective potential of singing to strengthen them will be researched by means of individual case observations and their agglutination into a single case experimental study.

Pilot study

May 2019 Basline survey and measurement (pre) of the intervention group.

Baseline survey via Fox trial finder of the control group (without intervention, optionally also patients with post-traumatic disorder without Parkinson's disease or other neurological disease)

June-July 2019 Intervention: 8 x 60min singing
group July 2019 Measurement (post)
November 2019 If necessary,

catamnesis preparation for the main

study from autumn 2019

Estimated schedule:

Winter 2018	Spring 2019	Summer 2019	Autum n 2019	Winter 2019	Spring 2020	Summer 2020
Literature Studies	Pilot studies	Pilot studies		Main study	Main study	
Application s	Elaboration of theory	Elaboration if necessary Publication-process	Elaboration if necessary Publication-process		Elaboration if necessary Publication-process	Elaboration if necessary Publication-process

2. Collection methods and procedures used Overview of planned procedures:

Pilot phase

Interventions	
Singing according to the concept of the Singing Hospitals e.V.	*www.singende-kranknhaeuser.de Publications by E.Wünnenberg (2017)*
Questionnaire survey	
Anamnesis form	Separate questionnaire for the examination of pre-treatments, access to the Singen, see appendix
PSI functional analysis of the Personality	https://impart.de/leistungen/testmodule/
Body Scales Questionnaire (BSQ)	http://stephenporges.com/index.php/publicationss/21-body-perception-questionnaires
United Parkinson Desaese Rating Scales UPDRS	http://www.neuromobil.ch/Uploads/Documents/0/UPDRS1.pdf
Freeze of Gait (FOG)	https://eprovide.mapi-trust.org/instruments/freezing-of-gait-questionnaire
Resilience Scale-RS 13	https://www.uniklinikum-jena.de/mpsy_media/Downloads/RESILIENZSKALA_LEPPERT2008.pdf
Visual analogue scale for the state of mind at the beginning End of the Intervention	*Own analogue scale, see appendix*

Psycho-physiological Measurement	
Heart Rate Variability Measurement	https://www.vnsanalyse.de/

Interventions	
Singing according to the concept of the singers Hospitals e.V.	*www.singende-kranknhaeuser.de* *Publications by E.Wünnenberg*
Optional: Safe and Sound Protocol, Stephen Porges	*https:www.traumaheilung.net/SSP-Safe-and-Sound-Protocol.pdf*
Sensorimotor training & Stochastic Resonance Training (SRT)	
Questionnaires	
Body Scales Questionnaire (BSQ)	http://stephenporges.com/index.php/publicationss/21-body-perception-questionnaires
PSI Functional Analysis of Personality	https://impart.de/leistungen/testmodule/
Resilience Scale-RS 13/RS-25	https://www.uniklinikum-jena.de/mpsy_media/Downloads/RESILIENZSKALA_LEPPERT2008.pdf
Optional: Structural analysis social behaviour (self-contact)	https://cip-medien.com/shop/sasb-die-strukturale-analyse-sozialen-behavioural/
Psycho-physiological measurement	
Heart Rate Variability Measurement, COMMIT	https://www.vnsanalyse.de/

1. Planned start and expected duration Spring 2019 - Summer 2020

In a second research step, an intervention study in the form of a randomized control study (possibly with a waiting group design), multiple baseline design or randomized subject design (main study) is to be carried out (corresponding application extension will be submitted to the ethics committee in the course of the pilot study).

D Selection of subjects

1. General information

Patients with diagnosed Parkinson's disease are in individual and group offers that complete stochastic resonance training (STR), education and an individual sports science training program at the Neurowerkstatt Pfungstadt (with different duration and frequency). All previous therapies (medication, physiotherapy) are documented and maintained. Recruitment is done via notice board and personal contact via Dr. M. Schwed, Neurowerkstatt Pfungstadt.

Control group will take the form of information letters to treating physicians, letters from support groups and Fox Trial Finder, and newspaper advertising.

2. Inclusion and exclusion criteria for subjects

Involvement in specialist treatment to check the state of health and medication

- Diagnosis of primary Parkinson's syndrome (G20.0-G20.2 according to ICD-10), possibly with comorbid psychological sequelae, but no other neurological disease, exclusion of dementia

- To describe the sample and check sample biases, patients diagnosed with "reactions to severe stress and adjustment disorders" (F43.0-F43.2 according to ICD-10), possibly with comorbid mental sequelae, but no persistent personality disorder (F 62) according to ICD-10, as well as subjects without a diagnosis of a physical illness or mental disorder, are included in the baseline survey.

3. Replacement of test subjects after admission to the research project (in the event of premature termination of participation)

There are no plans to replace test persons after they have been accepted into the research project.
Non-responders and dropout cases are documented.

4. Checking the state of health before and during the research project The state of health is checked by the treating specialists of the test subjects.

5. Termination criteria
- Withdrawal of the declaration of consent
- Subjectively perceived or clinically diagnosed mental or physical decompensation

E Statistical evaluation

Pilot studies: single-case diagnostic testing using non-overlap measures, nonparametric tests, randomization tests. Agglutination to a Single Case Experimental Study (Pospeschill & Siegel, 2018)

- Main study: Estimation of sample size according to MID Minimum Importent Difference, layer dimensions, Dispersion measures, Measures of correlations, T-test or covariance analysis with baseline data

F Ethical-legal aspects

1. Existing laws, regulations, guidelines

Guidelines of the Professional Association (Chamber of Psychotherapists BW)
Ethical guidelines of the initiative "Singing Hospitals e.V.

2. Cost-benefit analysis

Due to the step-by-step structure (pilot phase, main study), the hypotheses derived from recognized scientific theories and the choice of valid measuring instruments, a high gain in knowledge in relation to the personnel and financial effort involved is to be expected. Studies on relationship prevention or approaches based on the concept of salutogenesis/resilience increase patient competencies and reduce secondary diseases, so that the study meets the requirements of the steering group of the Future Forum Public Health (2017).

3. Insurance cover

Professional liability insurance of the experimenter, group liability insurance of the certified singing instructors via Initiative Singende Krankenhäuser e.V. or commissioning institutions of the singing offers

4. Preliminary tests

Study commissioned by the Singenden Krankenhausen e.V. by Kreutz, Böhm, Bossinger & Clift 2014/2015

5. Subject information/declaration of consent

Dear Ladies and Gentlemen, Dear singing enthusiasts and singing shy people!

I would like to personally invite you to participate in an innovative study to investigate the healing effects of singing in dealing with Parkinson's disease.
The study will start in January 2019 and will include participation in singing groups as well as accompanying interviews, questionnaire surveys and innovative psychovegetative treatment impulses.
The aim of the study is to show that there are starting points to counteract a vicious circle of physical/emotional torpor, loss of self-efficacy, contact and the experience of vitality. Instead of treatment in the classical sense, the focus here is on accessing and researching individual resources that determine the course of the disease compensate for deficits and stimulate neuroprotection and health prevention in a playful way.

The idea for this study was awarded a 10,000 euro prize by the Hilde Ulrichs Foundation for Parkinson's Research in September 2018. The study is also intended to contribute to the concern and mission of the non-profit international initiative "Singende Krankenhausen e.V." (see www.singende-krankenhaeuser.de). The initiative is committed to establishing health-promoting community singing offers in the health system. Health facilities are thus to become a place of encounter that shows the uniqueness, dignity and relationship orientation of people as an important contribution to treatment. There will also be a Koo https:// www.angela-brantzen.de/peration with the "neurowerkstatt" Pfungstadt (sports scientist Dr. M. Schwed, www.neurowerkstatt.de) and psychologist and certified singing leader Angela Brantzen (https:// www.angela-brantzen.de/).

The study is taking place as part of a doctorate under the supervision of Prof. Dr. Nicola Baumann and was approved by the Ethics Committee of the University of Trier. It includes various interventions and surveys/measurements, about which separate information is provided according to a random assignment. No study participant will receive a placebo or undergo any intervention (except for the control group of "healthy patients").
Your participation in the study is voluntary. You will therefore only be included in this examination if you declare your consent in writing. If you do not want to participate in the study or withdraw from it later, you will not suffer any disadvantages. At any time, you can discontinue the study or withdraw the use of your anonymized data without personal disadvantage. By participating in the study, you have the right to a 30-50 minute personal final interview by the head of the study, Elke Wünnenberg.

The following text is intended to explain the goals and the process. An investigator will then conduct the information interview with you. Please do not hesitate to address any points that are unclear to you. You will then be given sufficient time to think about your participation.
For further information, please refer to the appendix or the homepage www.singende-krankenhaeuser.de

Singing is medicine – what is the truth of this statement?
"Singing as medicine" is first and foremost a slogan to draw the attention of all those involved in the health care system, especially patients and their practitioners, to the potential of singing. The most important difference to a remedy is that when singing, the effect is not based on a substance that is administered from the outside. Singing appeals to people in their essence and activates inner self-healing powers. In addition, singing to spontaneously sing along can be contagious - so it has a comparable effect to a "health pathogen".
Does it matter what I sing?

What is more important is the attitude of how you sing. In this study, simple, life-affirming songs of all cultures are sung together, which do not create any pressure to perform. No previous musical knowledge is necessary.

How does singing affect health in concrete terms?
Singing is like a "hotline" to the autonomic nervous system and can balance it, especially in times of crisis, when chronic stress reactions, inadequate coping strategies and withdrawal behaviors create a vicious circle. Through calm, flowing, contemplative songs, the parasympathetic nervous system discharges excess energy and relaxes the body, enabling inner contemplation and flower life. Through faster structured-rhythmic songs, the sympathetic nervous system charges, energizes the body, and the energy of action, courage and drive are strengthened.

How do Parkinson's sufferers benefit from this?
Singing can help to experience that drive, contact, joy of movement and community are possible despite dopamine deficits. Singing gives playful access, which one otherwise often tries to achieve through training and self-discipline. Music has a potential for self-soothing and self-motivation – especially in active singing. The most important factor is that singing through breath and sound can relieve physical and emotional rigidity and strengthen self-efficacy and community experience.

Is this scientifically proven?
An overview of the state of scientific knowledge is provided by Gunther Kreutz's book "Why Singing Makes You Happy" and the publication of the Initiative Singende Krankenhausen e.V. "Singing as a healing power. The potential of singing for the whole world is groundbreaking in the field of singing and attachment research. A review article on all studies on singing with Parkinson's patients by Barnish et al. 2018 found evidence of therapeutic potential that needs to be explored in more detail with regard to communication, cognitive status, motor skills and quality of life.

Director of Studies and Contact Person
Elke Wünnenberg (Psychologist. Psychotherapist, certified music educator),

Since its foundation in 2009 member of the Singenden Krankenhausen e.V., since 2012 on the

board of directors, since 2016 chairwoman of the board. Award winner of the Hilde Ulrichs

Foundation for Parkinson's Research 2018.

Please contact: elkewuennenberg@web.de; Phone: 0152-34531920

With my signature, I agree to be contacted by the director of studies.

NAME	CONTACT DETAILS<br>(e-mail, phone)	Signature (Consent th e Contact)

1. Confidentiality of Subject Data

In the pilot phase, data is stored pseudonymously by assigning a patient ID, which is only accessible to the study leader. The first 2 first letters of the first and last name are encrypted via a randomly generated coding system (A becomes I, B becomes H....Z becomes M). This encryption key will be kept locked and will not be passed on to third parties. All questionnaires and measurements are provided with this coding and not with the name of the subject. third parties (supervisor, cooperation heart rate variability, EOS test system) receive the data only pseudonymized.

The data is stored and encrypted on a private business server/private cloud with backup function via Amazon). Only the Director of Studies has the right of access. After completion of the study, the data will be stored on the server of the University of Trier, Faculty I, Department of Differential Psychology. The test subjects are given a contact person to whom they can request the deletion of their data at any time. According to the rules for safeguarding good scientific practice, the data is generally only destroyed after 10 years, even if it is not used.

Informed consent: A declaration of consent for data collection, data processing and publication at specialist conferences, journals is obtained in writing from the test persons in the data protection declaration of the patient consent declaration after an information event. At the end of the study, there will be a 30-50 personal debriefing by the study leader with each subject. With the post-measurement, written consent is obtained from the test subjects and explicit permission to use the collected data as a reference date for future studies by the study leader.
According to the rules for safeguarding good scientific practice, this data can only be destroyed after 10 years.

2. Data Capture

Paper-Pencil :

- Body Experience Questionnaire (Porges)
- Resilience questionnaire

computer-aided

- System Development Diagnostics
- Heart rate variability measurement via VNS analysis, Commit GmbH www.commitgmbh.de

3. *Insurance*

   It is not considered necessary to take out no-fault insurance for test subjects.

Literature used:

Adamek, K. (1996). *Singing as a life coach. On Empirical and Theory of Coping with Everyday Life*. Plea for a "Renewed Everyday Culture of Singing". Münster: Waxmann.

Barnish J, Atkinson RA, Barran SM, Barnish MS. (2016). Potential Benefit of Singing for People with Parkinson's Disease: A Systematic Review. J Parkinsons Dis. 3; 6(3):473-84. doi: 10.3233/JPD-160837.

Blechert, J., Michael, T., Grossman, P., Lajtman, M., & Wilhelm, F. H. (2007). Autonomic and respiratory characteristics of posttraumatic stress disorder and panic disorder. *Psychosomatic Medicine*, *69*(9), 935–943. https://doi.org/10.1097/PSY.0b013e31815a8f6b

Bossinger, W. (2006). *The healing power of singing. From the origins to modern findings about the social and health-promoting effects of singing*. Battweiler: Dreamtime.

Briegel, W., Walter, T., Schimek, M., Knapp, D., & Büssing, R. (2015). Parent-Child Interaction Therapy in in-room coaching. Results of a first German case study. *Childhood and Development*, *24*(1), 47–54. https://doi.org/10.1026/0942-5403/a000158

Cho, K.-H., Kim, T.-H., Kwon, S., Jung, W.-S., Moon, S.-K., Ko, C.-N., ... Chung, E. K. (2018). Complementary and alternative medicine for idiopathic Parkinson's disease: An evidence-based clinical practice guideline. *Frontiers in Aging Neuroscience*, *10*. https://doi.org/10.3389/fnagi.2018.00323

Combs HL, Garcia-Willingham NE, Berry DTR, van Horne CG, Segerstrom SC. (2018). Psychological functioning in Parkinson's disease post-deep brain stimulation: Self-regulation and executive functioning. J Psychosom Res.; 111:42-49. doi: 10.1016/j.jpsychores.2018.05.007. Epub 2018 May 21.

Christian T. Haas, Stephan Turbanski, Dietmar Schmidtbleicher (2006). How deliberate disorder in training ensures order in movement Random oscillations have an effect on muscle and nerve cells. Sensorimotor Training & Stochastic Resonance Training (SRT). Forschungs Frankfurt 4, 19-25.

Cossu, G., Rinaldi, R., & Colosimo, C. (2018). The rise and fall of impulse control behavior disorders. *Parkinsonism & Related Disorders*, *46*(Suppl 1), S24–S29. https://doi.org/10.1016/j.parkreldis.2017.07.030

Dale, L. P., Shaikh, S. K., Fasciano, L. C., Watorek, V. D., Heilman, K. J., & Porges, S. W. (2017). College Females With Maltreatment Histories Have Atypical Autonomic Regulation and Poor Psychological Wellbeing. *Psychological Trauma: Theory, Research, Practice, andPolicy*. Advance online publication. http://dx.doi.org/10.1037/tra0000342

De Bock, F., Geene, R., Hoffmann, W., Stang, A. (2017): Handout from the steering group of the Future Forum Public Health for all actors involved in prevention in practice and politics Submitted by an ad hoc working group by Zukunftsforum Public Health, Berlin, Available at: https://zukunftsforum-public-health.de/wp-content /uploads/2018/01/2017_12_Handreichung_Verh%C3%A4ltnispr%C3%A4vention_Zukunftsforum.pdf

Devereaux (2017) An Interview with Dr. Stephen W. Porges- Am J Dance Ther 39:27–35. DOI 10.1007/s10465-017-9252-6

Drury, R. (2014). Wearable biosensor systems and resilience: a perfect storm in health care? Review Article. *Frontiers in Psychology*, 5, 853, 1-5. DOI: 10.3389/fpsyg.2014.00853

Faherty, C. J., Shepherd, K. R., Herasimtschuk, A., & Smeyne, R. J. (2005). Environmental enrichment in adulthood eliminates neuronal death in experimental Parkinsonism. *Molecular Brain Research*, *134*(1), 170–179. https://doi.org

Fahn, S. (2018). The 200-year journey of Parkinson disease: Reflecting on the past and looking towards the future. Parkinsonism and Related Disorders 46 (2018) S1eS

Faul, F., Erdfelder, E., Lang, A.-G., & Buchner, A. (2007). G*Power 3: A flexible statistical power analysis program for the social, behavioral, and biomedical sciences. Behavior Research Methods, 39, 175-191. Download PDF

Green KT1, Dennis PA2, Neal LC3, Hobkirk AL4, Hicks TA4, Watkins LL4, Hayano J5, Sherwood A4, Calhoun PS6, Beckham JC6. (2016). Exploring the relationship between posttraumatic stress disorder symptoms and momentary heart rate variability. J Psychosom Res. 2016 Mar;82:31-4. doi: 10.1016/j.jpsychores.2016.01.003. Epub 2016 Jan 13.

Flores Alves Dos Santos, J., Tezenas du Montcel, S., Gargiulo, M., Behar, C., Montel, S., Hergueta, T., … Welter, M.-L. (2017). Tackling psychosocial maladjustment in Parkinson's disease patients following subthalamic deep-brain stimulation: A randomised clinical trial. *PLoS ONE*, *12*(4).

Garlovsky, J. K., Overton, P. G., & Simpson, J. (2016). Psychological predictors of anxiety and depression in Parkinson's disease: A systematic review. *Journal of Clinical Psychology*, *72*(10), 979–998. https://doi.org/10.1002/jclp.22308

Hamilton, J. & Alloy, L. (2016): Atypical reactivity of heart rate variability to stress and depression across development: Systematic review of the literature and directions for future research. *Clinical Psychology Review,* 50, 67–79. DOI: 10.1016/j.cpr.2016.09.003. Epub 2016 Sep 20

Hautzinger M. (1994). Action control in the context of psychopathological disorders. In J. Kuhl & J.Beckmann (Hrsg.), Volition and personality: Action versus state orientation (S.209-215). Seattle: Hogrefe.

Hawkes, C.H., Del Tredici, K., Braak, H. (2010). A timeline for Parkinson's disease. Parkinsonism and Related Disorders 16 (2010) 79–84

Heller, L. & Lapierre, A. (2013). *Healing developmental trauma. Solving Old Survival Strategies – Strengthening Self-Regulation and Relationship Skills – The Neuroaffective Relationship Model for Trauma Healing NARM.* Munich: Kösel.

Holt-Lunstad, J., Smith, T. & Layton, J. (2010). Social Relationships and Mortality Risk: A Meta-analytic Review. *PLoS Medicine, 7* (7). https://doi.org/10.1371/journal.pmed.1000316.

Holzman JB, Bridgett DJ. Heart rate variability indices as bio-markers of top-down self-regulatory mechanisms: A meta-analytic review. Neurosci Biobehav Rev. 2017 Mar; 74(Pt A):233-255. doi: 10.1016/j.neubiorev.2016.12.032. Epub 2017 Jan 3.

Jungen, M. Personal conversations and unpublished documents from resilience trainings and projects in which organizational resilience concepts were realized.

Kang, J & Scholp, A. & Jiang, J. (2017). A Review of the Physiological Effects and Mechanisms of Singing. *Journal of Voice*. DOI: 10.1016/j.jvoice.2017.07.008.

Kempa, A.H, Julian Koenigc, Julian F. Thayerd (2017). From psychological moments to mortality: A multidisciplinary synthesis on heart rate variability spanning the continuum of time. Neuroscience and Biobehavioral Reviews 83, 547–567

Klaissle, P., Lesemann, A., Huehnchen, P., Hermann, A., Storch, A., & Steiner, B. (2012). Physical activity and environmental enrichment regulate the generation of neural precursors in the adult mouse substantia nigra in a dopamine-dependent manner. *BMC Neuroscience*, *13*. Retrieved from http://www.redi-bw.de/db/ebsco.php/search.ebscohost.com/login.aspx%3fdirect%3dtrue%26db%3dpsyh%26AN%3d2014-50040-001%26site%3dehost-live

Kreutz, G. (2014). *Why singing makes you happy*. Giessen: Psychosozial-Verlag.

Kreutz, G., Clift, S., Böhm, K. & Bossinger, W. (2017). *Singing hospitals from the point of view of singing group leaders.* In E. Wünnenberg (ed.), Singing as a Healing Power. The potential of singing for the healthcare system. Grundlagen-Praxisfelder-Perspektiven, 2. Aufl., 164-190, Bad Waldsee: Selbstverlag.

Kuhl, J. (2001). *Motivation and personality. Interaction of Psychic Systems.* Göttingen: Hogrefe.

Lenka, A., Hegde, S., Arumugham, S. S., & Pal, P. K. (2017). Pattern of cognitive impairment in patients with Parkinson's disease and psychosis: A critical review. *Parkinsonism & Related Disorders*, *37*, 11–18. https://doi.org/10.1016/j.parkreldis.2016.12.025

Leppert, Koch, Bräher & Strauss (2008). The resilience scale. Verification of the long form RS-25 and a short form RS-13. Klin. Diagnostics and Evaluation, 1, 226-243

Lidstone SC (2014). Great expectations: the placebo effect in Parkinson's disease. Handb Exp Pharmacol. 2014;225:139-47. doi: 10.1007/978-3-662-44519-8_8.

Maetzler W1, Liepelt I, Berg D. (2009). Progression of Parkinson's disease in the clinical phase: potential markers. Lancet Neurol. 2009 Dec; 8(12):1158-71. doi: 10.1016/S1474-4422(09)70291-1.

Macht, M., & Ellgring, H. (1999). Behavioral analysis of the freezing phenomenon in Parkinson's disease: a case study. *Journal of Behavior Therapy and Experimental Psychiatry*, *30*, 241–247. https://doi.org/10.1016/S0005-7916(99)00021-X

Marlysa B. Sullivan, Matt Erb, Laura Schmalzl, Steffany Moonaz,

Marques, A., Durif, F., & Fernagut, P.-O. (2018). Impulse control disorders in Parkinson's disease. *Journal of Neural Transmission*, *125*(8), 1299–1312. https://doi.org/10.1007/s00702-018-1870-8

Muehsam. D. Lutgendorf, S., Mills, P.J., Rickhi, B., Chevalier, G., Bat, N. , Chopra, D., Gurfein, B. (2017). The embodied mind: A review on functional genomic and neurological correlates of mind-body therapies. Neuroscience & Biobehavioral Reviews, 73, 165-181. https://doi.org/10.1016/j.neubiorev.2016.12.027

Nithianantharajah, J., & Hannan, A. J. (2006). Enriched environments, experience-dependent plasticity and disorders of the nervous system. *Nature Reviews Neuroscience*, *7*(9), 697–709. https://doi.org/10.1038/nrn1970

Park, J. E., Lee, J. Y., Kang, S.-H., Choi, J. H., Kim, T. Y., So, H. S., & Yoon, I.-Y. (2017). Heart rate variability of chronic posttraumatic stress disorder in the Korean veterans. *Psychiatry Research*, *255*, 72–77. https://doi.org/10.1016/j.psychres.2017.05.011

Petkus, Andrew J., J. Vincent Filoteo, Green, K. T., Dennis, P. A., Neal, L. C., Hobkirk, A. L., Hicks, T. A., Watkins, L. L., Beckham, J. C. (2016). Exploring the relationship between posttraumatic stress disorder symptoms and momentary heart rate variability. *Journal of Psychosomatic Research*, *82*, 31–34. https://doi.org/10.1016/j.jpsychores.2016.01.003

Porges S.W. (1995). Orienting in a defensive world: mammalian modifications of our evolutionary heritage. A Polyvagal Theory. Psychophysiology. 32(4):301-18.

Porges, S.W. (2010). *The Polyvagal Theory: Neurophysiological Foundations of Therapy*. Paderborn: Junfermann.

Porges, S.W. (2017). *The polyvagal theory and the search for security. Trauma treatment, social commitment and bonding*. Conversations and Reflections on the Polyvagal Theory. Lichtenau: Probst.

Porges, S.W. (2007). A phylogenetic journey through the vague and ambiguous Xth cranial nerve: a commentary on contemporary heart rate variability research. Biol Psychol.; 74(2):301-7. DOI:10.1016/j.biopsycho.2006.08.007

Pospeschill, M. & Siegel, R. (2018) Methods for Clinical Research and Diagnostic Practice. Heidelberg: Springer-Verlag.

Rönnau-Böse, M. (2014): Resilience. "3rd, updated edition." Munich, Basel: Ernst Reinhardt.

Rönnau-Böse, M., & Fröhlich-Gildhoff, K. (2015). Resilience and resilience promotion via the Life span. Kohlhammer Verlag

Rüegg (2001): Psychosomatics, Psychotherapy and the Brain. Neuronal plasticity as the basis of biopsychosocial medicine. Stuttgart: Schattauer.

Salsone M, Vescio B, Fratto A, Sturniolo M, Arabia G, Gambardella A, Quattrone A. (2016). Cardiac sympathetic index identifies patients with Parkinson's disease and REM behavior disorder.Parkinsonism Relat Disord. 26:62-6. doi: 10.1016/j.parkreldis.2016.03.004. Epub 2016 Mar 10.

Schiavio, A. & Altenmüller, E. (2015): Exploring music-based rehabilitation for Parkinsonism through embodied cognitive science. Frontiers in Neurology. https://doi.org/10.3389/fneur.2015.00217

Schiepek, G. & Matschi, B. (2013). Resource Capture in the Therapeutic Process. Presentation, promotion and sustainable use. Psychotherapy in Dialogue, 14:1, 56-61. DOI: 10.1055/ s-0033-1337098

Seligowski, A. V., Lee, D. J., Bardeen, J. R., & Orcutt, H. K. (2015). Emotion regulation and posttraumatic stress symptoms: A meta-analysis. *Cognitive Behaviour Therapy*, *44*(2), 87–102. https://doi.org/10.1080/16506073.2014.980753

Sullivan, M.,Erb, M. Schmalzl, L.,Moonaz, S. Noggle, J. Taylor & Porges, S.W.(2018). The Convergence of Traditional Wisdom and Contemporary Neuroscience for Self-Regulation and Resilience, Front. Hum. Neurosci. https://doi.org/10.3389/fnhum.2018.00067

Strano S, Fanciulli A, Rizzo M, Marinelli P, Palange P, Tiple D, De Vincentis G, Calcagnini G, Censi F, Meco G, Colosimo C. (2016). Cardiovascular dysfunction in untreated Parkinson's disease: A multi-modality assessment. J Neurol Sci.; 370:251-255. doi: 10.1016/j.jns.2016.09.036. Epub 2016 Sep 21.

Solla P1, Cadeddu C2, Cannas A1, Deidda M3, Mura N3, Mercuro G3, Marrosu F1.(2015). Heart rate variability shows different cardiovascular modulation in Parkinson's disease patients with tremor dominant subtype compared to those with akinetic rigid dominant subtype. J Neural Transm (Vienna).122(10):1441-6. doi: 10.1007/s00702-015-1393-5. Epub 2015 Mar 24.

Tass, P. A., Qin, L., Hauptmann, C., Dovero, S., Bezard, E., Boraud, T., & Meissner, W. G. (2012). Coordinated reset has sustained aftereffects in Parkinsonian monkeys. *Annals of Neurology*, *72*(5), 816–820. https://doi.org/10.1002/ana.23663

Thiriet, N., Amar, L., Toussay, X., Lardeux, V., Ladenheim, B., Becker, K. G., ... Jaber, M. (2008). Environmental enrichment during adolescence regulates gene expression in the striatum of mice. *Brain Research*, *1222*, 31–41. https://doi.org/10.1016/j.brainres.2008.05.030

Trojano, L., & Papagno, C. (2018). Cognitive and behavioral disorders in Parkinson's disease: An update II: Behavioral disorders. *Neurological Sciences*, *39*(1), 53–61. https://doi.org/10.1007/s10072-017-3155-7

Trösken A.K. (2010). The Bernese Resource Inventory. Resource Potentials and Resource Realization from a Consistency Theory Inaugural Dissertation of the Faculty of Philosophy and History of the University of Bern on the Acquisition of the Doctorate submitted by Federal Republic of Germany Institute of Psychology University of Bern

Tugade, M. M., & Fredrickson, B. L. (2004). Resilient Individuals Use Positive Emotions to Bounce Back From Negative Emotional Experiences. *Journal of Personality and Social Psychology*, *86*(2), 320–333. https://doi.org/10.1037/0022-3514.86.2.320

Uem, J. M. T., Marinus, J., Canning, C., van Lummel, R., Dodel, R., Liepelt-Scarfone, I., ... Maetzler, W. (2016). Health-Related Quality of Life in patients with Parkinson's disease—A systematic review based on the ICF model. *Neuroscience and Biobehavioral Reviews*, *61*, 26–34. https://doi.org/10.1016/j.neubiorev.2015.11.014

Walkera, F., Pfingst, K., Carnevalic, L., Sgoifoc, A. & Nalivaikoa, E. (2017). In the search for integrative biomarker of resilience to psychological stress, *Neuroscience and Biobehavioral Reviews*, 74, 310–320, DOI: 10.1016/j.neubiorev.2016.05.003. Epub 2016 May 11

Wünnenberg, E. (2017a). *To be related to singing. Draft of a Theory & Methodology of Healing Singing for Disease Management and Health Care,* In E. Wünnenberg (Ed.), Singing as a Healing Power. The potential of singing for the healthcare system. Grundlagen- Praxisfelder-Perspektiven, 2. Aufl., 76-116, Bad Waldsee: Selbstverlag.

Wünnenberg, E. (2017b). *Singing & Resilience. From Singing to Being. How Singing Strengthens Us: Self-Regulation in a Sounding Way,* In E. Wünnenberg (Ed.), Singing as a Healing Power. The potential of singing for the healthcare system. Grundlagen-Praxisfelder-Perspektiven, 2. Aufl., 118-148, Bad Waldsee: Selbstverlag.

Wünnenberg (to be published in spring 2019). Singing Hospitals e.V. In; Decker-Voigt, H. & Weymann (eds): Musiktherapie-Lexikon. Göttingen: Hogrefe.

Wünnenberg (to be published in spring 2019). Self-regulation – adaptive development – crisis competences. In; Decker-Voigt, H. & Weymann (eds): Musiktherapie-Lexikon. Göttingen: Hogrefe.

Xian-Si Zeng*, Wen-Shuo Geng, Jin-Jing Jia*, Lei Chen and Peng-Peng Zhang (2018).Cellular and Molecular Basis of Neurodegeneration in Parkinson Disease, doi: 10.3389/fnagi.2018.00109

Xie, C.-L., Wang, X.-D., Chen, J., Lin, H.-Z., Chen, Y.-H., Pan, J.-L., & Wang, W.-W. (2015). A systematic review and meta-analysis of cognitive behavioral and psychodynamic therapy for depression in Parkinson's disease patients. *Neurological Sciences*, *36*(6), 833–843. https://doi.org/10.1007/s10072-015-2118-0
